# Quantitative Homogenization for the Obstacle Problem and Its Free Boundary

**DOI:** 10.1007/s00205-024-02015-6

**Published:** 2024-08-18

**Authors:** Gohar Aleksanyan, Tuomo Kuusi

**Affiliations:** Department of Mathematics and Statistics, University of Helsinki, Helsinki, Finland

## Abstract

In this manuscript we prove quantitative homogenization results for the obstacle problem with bounded measurable coefficients. As a consequence, large-scale regularity results both for the solution and the free boundary for the heterogeneous obstacle problem are derived.

## Introduction

In this manuscript we investigate the stochastic homogenization of the normalized obstacle problem. Our main goal is to prove regularity results for the solution and for the free boundary of the following heterogenous obstacle problem

1.1 $$\begin{aligned} \nabla \cdot (\textbf{a}^\varepsilon \nabla u^\varepsilon )=f \chi _{\{ u^\varepsilon >0\} } \end{aligned}$$

in a given Lipschitz domain $U \subseteq \mathbb {R}^d$. Here  *f* is a positive function and $\textbf{a}(\cdot )$ is a uniformly elliptic measurable coefficient field (see 1.14) and we denote $\textbf{a}^\varepsilon (x) := \textbf{a}(\frac{x}{\varepsilon })$ for a small parameter $\varepsilon >0$. We shall clarify all of our assumptions below.

Let us first discuss the connection of (1.1) to the well-known classical obstacle problem. Let $\varphi \in H^{1}(U)$ be the given function, called an *obstacle*, and let $g \in H^{1}(U)$ correspond to the boundary data, such that $g \ge \varphi$ on $\partial U$. Consider the energy

1.2 $$\begin{aligned} J[v, \textbf{a}^\varepsilon , U]:= \frac{1}{2} \int _U \textbf{a}^\varepsilon ( x )\nabla v(x) \cdot \nabla v(x) \textrm{d}x, \end{aligned}$$

and the minimization problem

1.3 $$\begin{aligned} \min _{v \in \mathcal {K}(\varphi , g, U)} J[v, \textbf{a}^\varepsilon , U], \text { where} \quad \mathcal {K}(\varphi , g, U) := \big \{ v \in g + H_0^1(U) \, : \, v \ge \varphi \big \}. \end{aligned}$$

Notice that the convex set $\mathcal {K}(\varphi , g, U)$ is not empty, since we assume that $g \ge \varphi$ on $\partial U$. The existence and uniqueness of the minimizer to (1.3) can be shown via direct methods in the calculus of variation, see for example, [29]. The minimizer $v^\varepsilon$ is called * the solution to the obstacle problem* (1.3) and it satisfies the following system of inequalities in a weak sense:

1.4 $$\begin{aligned} \left\{ \begin{aligned}&-\nabla \cdot \textbf{a}^\varepsilon \nabla v^\varepsilon \ge 0  &   \text{ in } U \\&-\nabla \cdot \textbf{a}^\varepsilon \nabla v^\varepsilon = 0  &   \text{ in } \{ v^\varepsilon > \varphi \} \cap U \\&v^\varepsilon \ge \varphi  &   \text{ in } U \\&v^\varepsilon = g  &   \text{ on } \partial U . \end{aligned} \right. \end{aligned}$$

The set $\varOmega (v^\varepsilon ):= U \cap \{ v^\varepsilon >\varphi \}$ is called the *non-coincidence set*, and $\varGamma (v^\varepsilon ):=\partial \varOmega ( v^\varepsilon ) \cap U$ is called the *the free boundary*. The *coincidence* or *contact set* is denoted by $\varLambda (v^\varepsilon ):=\{ v^\varepsilon \equiv \varphi \}$. By (1.4), the minimizer $v^\varepsilon$ is a weak supersolution over the whole domain *U* and $v^\varepsilon$ is $\textbf{a}^\varepsilon$-harmonic in the non-coincidence set. Observe that the free boundary $\varGamma (v^\varepsilon )$ depends on the solution $v^\varepsilon$ and is not known a priori. Moreover, we see that $\nabla \cdot (\textbf{a}^\varepsilon \nabla v^\varepsilon ) = \nabla \cdot (\textbf{a}^\varepsilon \nabla \varphi )$ in the interior of the set $\{v^\varepsilon =\varphi \}$, while $\nabla \cdot (\textbf{a}^\varepsilon \nabla v^\varepsilon ) = 0$ in the non-coincidence set.

The obstacle problem has numerous applications, from option pricing in financial mathematics to tumor growth problems in biology. Free boundary problems of similar nature appear in mathematical modeling of various problems in physics and life sciences. Our contribution is to study these type of problems in heterogeneous, possibly random, medium.

When investigating free boundary problems, we ask two main questions. First, what is the optimal regularity of the solution? Second, what can we say about the regularity of the free boundary? In the context of homogenization, we additionally ask the question of whether we can approximate the solution $v^\varepsilon$ with a solution to the corresponding homogenized obstacle problem as $\varepsilon \rightarrow 0$,1.5where  is a symmetric positive matrix, the *homogenized matrix*, which can be identified under suitable deterministic or stochastic *ergodicity* assumptions. The exact assumptions on the coefficient field $\textbf{a}$ will be discussed in Sect. 2.3. The subsequent question is if and when we can prove homogenization of the corresponding free boundaries?

Now let us proceed to more detailed discussion of the problem under investigation, (1.1). Let $\omega ^\varepsilon :=v^\varepsilon -\varphi \ge 0$. Then we derive from (1.4) that $\omega ^\varepsilon \ge 0$ satisfies variational inequality in a weak sense,

$$\begin{aligned} \nabla \cdot ( \textbf{a}^\varepsilon \nabla \omega ^\varepsilon ) = - \nabla \cdot ( \textbf{a}^\varepsilon \nabla \varphi ) \chi _{\{V^\varepsilon >0\}} \,, \end{aligned}$$

with corresponding boundary conditions. In order to be able to control the growth of the solution $\omega ^\varepsilon$ at free boundary points, we need some control over $\nabla \cdot ( \textbf{a}^\varepsilon \nabla \varphi )$, which oscillates rapidly as $\varepsilon \rightarrow 0+$ and is, in general, merely a distribution in $H^{-1}(U)$. To remedy this, we will consider a heterogeneous obstacle instead of the original one. For given $\varphi \in H^{1}(U)$, define $\varphi ^\varepsilon \in \varphi + H_0^1(U)$ to be the solution of1.6Via integration by parts the energy in (1.2) can be rewritten as

$$\begin{aligned}&J[v;\textbf{a}^\varepsilon ,U] = \int _U \left( \frac{1}{2} \textbf{a}^\varepsilon \nabla (v-\varphi ^\varepsilon ) \cdot \nabla (v-\varphi ^\varepsilon ) + (v-\varphi ^\varepsilon ) (-\nabla \cdot \textbf{a}^\varepsilon \nabla \varphi ^\varepsilon )\right) \\&\quad + \int _U \left( (g - \varphi ^\varepsilon )(-\nabla \cdot \textbf{a}^\varepsilon \nabla \varphi ^\varepsilon ) + \textbf{a}^\varepsilon \nabla \varphi ^\varepsilon \cdot \nabla (g - \varphi ^\varepsilon ) + \frac{1}{2} \textbf{a}^\varepsilon \nabla \varphi ^\varepsilon \cdot \nabla \varphi ^\varepsilon \right) \,, \end{aligned}$$

where the second integral on the right is null-Lagrangian for fixed obstacle and boundary data. Let $u^\varepsilon := v^\varepsilon -\varphi ^\varepsilon \ge 0$ , then $u^\varepsilon$ is the minimizer of the first integral on the right, with the boundary data $g-\varphi$, and it satisfies (1.1). Similarly, $u: =v-\varphi \ge 0$ is the minimizer of the energyand it solves1.7The corresponding free boundaries for $u^\varepsilon$ and *u* are defined as follows:

1.8 $$\begin{aligned} \varGamma (u^\varepsilon ) =\partial \{ u^\varepsilon>0\} \cap U \quad \text{ and } \quad \varGamma (u) = \partial \{ u >0\} \cap U \,. \end{aligned}$$

The coincidence sets are denoted by

1.9 $$\begin{aligned} \varLambda (u^\varepsilon ) = \{ u^\varepsilon = 0\} \cap U \quad \text{ and } \quad \varLambda (u) = \{ u = 0\} \cap U \,. \end{aligned}$$

In order to obtain and large-scale regularity results for the solutions $u^\varepsilon$ and *u* and for corresponding free boundaries $\varGamma (u^\varepsilon )$ and $\varGamma (u)$, we need to impose more assumptions on *f*, such as uniform positivity and Hölder continuity. If we allow *f* to be negative in a subset $V \subseteq U$, then $V \cap \varGamma =\emptyset$ by a comparison principle. As a matter of fact, in Sect. 5 we simplify even further and take *f* to be a positive constant $\lambda$. There are counterexamples, showing that if we allow $f=0$ or *f* to be merely continuous, even when $\textbf{a}$ is a constant matrix, we cannot expect regularity of the free boundary, see [14] for further discussion. The normalization described above, and the assumptions on *f* are widely used in the context of free boundary problems. In fact in most of the literature $f \equiv 1$, since the more general situation can be addressed via stability properties of the free boundary; see [14] for details.

Now, let us make a very important observation: The solution to the original obstacle problem (1.3), denoted now by $v^\varepsilon _\varphi$, is close to the solution of the obstacle problem with the new obstacle $\varphi ^\varepsilon$. Indeed, comparison principle yields that

1.10 $$\begin{aligned} \left\| v^\varepsilon _\varphi - v^\varepsilon \right\| _{L^\infty } \le \left\| \varphi ^\varepsilon - \varphi \right\| _{L^\infty } = O(\varepsilon ), \end{aligned}$$

where the smallness of $\left\| \varphi ^\varepsilon - \varphi \right\| _{L^\infty }$ follows from the homogenization theory, see Lemmas 3.1 and 3.2 for details. Furthermore, the above estimate suggests that the homogenization of solutions of the normalized obstacle problem implies homogenization of solutions to the original obstacle problem (1.3), with a fixed obstacle. By the triangle inequality we have that

$$\begin{aligned} \left\| v^\varepsilon _\varphi - v \right\| _{L^\infty }&\le \left\| u^\varepsilon - u \right\| _{L^\infty } +2 \left\| \varphi ^\varepsilon -\varphi \right\| _{L^\infty } , \end{aligned}$$

where $u^\varepsilon$ and *u* satisfy  (1.1) and (1.7) respectively. Consequently, it suffices to investigate the homogenization of solutions of the normalized obstacle problem (1.1).

**Fig. 1:**
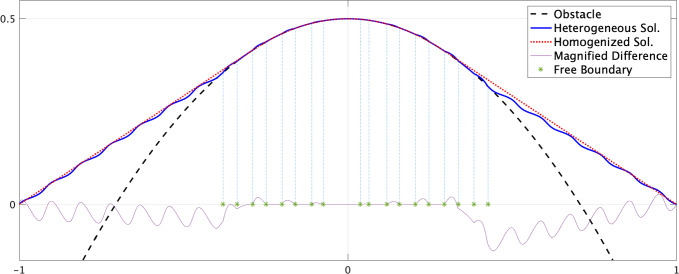
Given obstacle $\varphi = \frac{1}{2} - x^2$, there are plotted both heterogeneous and homogenized solutions, together with $\varphi$ and the free boundary. Also the difference, magnified by a factor of 6, between heterogeneous and homogenized solutions is plotted

The primary reason for focusing our attention on problem (1.1) is that homogenization of the free boundary does not hold when the obstacle is fixed due to wild oscillations of $\textbf{a}^\varepsilon$ for small $\varepsilon$ even if $\textbf{a}$ is smooth. This can be seen already in 1D-examples as demonstrated in Appendix A. We actually give two examples in the appendix. Example 2 shows that even if we have a fixed strictly concave smooth obstacle $\varphi$, then we may not have homogenization of the free boundary due to rapid oscillations of $\nabla \cdot (\textbf{a}^\varepsilon \nabla \varphi )~$as $\varepsilon \rightarrow 0+$, see Fig. 1 for numerical illustration. On the contrary, Example 1 shows that the homogenization of the solution and of the free boundary holds for the normalized obstacle problem. Letting $v^ \varepsilon \ge 0$ and $v \ge 0$, solve (1.1) and (1.7) respectively, with $f \equiv 1$ in the interval (0, 4), and letting $\alpha ^\varepsilon$ and $\alpha$ be the free boundary points for $v^\varepsilon$ and *v*, we show that then $v^\varepsilon \rightarrow v$ and $\alpha ^\varepsilon \rightarrow \alpha$.

Let us summarize the problem under investigation for the rest of the manuscript. Let $$ f \in L^q$$, $$q >d/2$$, $$ f >0$$ a.e., and consider the minimizer of the convex functional

1.11 $$\begin{aligned} I[u; \textbf{a}^\varepsilon , f, U]:= \int _U \frac{1}{2}\nabla u(x) \cdot \textbf{a}\left( \tfrac{x}{\varepsilon } \right) \nabla u(x) +f(x ) u(x)\textrm{d}x \end{aligned}$$

over  $\mathcal {K}^+(g, U):=\{ u \in H^{1}(U), ~v \ge 0 \text { in }~U,~ u =g >0 \text { on }~\partial U \}$. There is a unique minimizer $u^\varepsilon$, which we call the *heterogeneous solution* to the normalized obstacle problem, and it satisfies (1.1).

Let  *u* be the minimizer of the corresponding convex functional with the homogenized matrix  ( see the next subsection for the definition of the homogenized matrix )1.12over $u \in \mathcal {K}^+(g, U)$. Then *u* is called the *homogenized solution* to the normalized obstacle problem and it satisfies (1.7).

Concerning the regularity of the minimizer $u^\varepsilon$, for merely measurable coefficients, the solution is Hölder regular by De Giorgi-Nash-Moser type arguments, see for example [30]. If coefficients are $C^{0,\alpha }$ for some $\alpha \in (0,1]$, then the minimizer is $C^{1,\alpha }$-regular. When the coefficient matrix $\textbf{a}$ is constant (or sufficiently smooth), both the regularity of the solution and of the free boundary are well understood. The optimal regularity of solutions is due to Frehse [28]. When *f* is uniformly positive and Hölder continuous, the free boundary regularity is fully investigated by Caffarelli [18, 20], see also some recent contributions such as [24, 25]. The more general case, when *f* is Dini-continuous, the regularity of the free boundary is derived in see [14].

The obstacle problem with sufficiently regular variable coefficients have been studied using energy methods, see for example [27, 40]. However, there are more challenges appearing when the coefficients are merely measurable. Blank, jointly with collaborators, studied free boundary problems with rough coefficients, [15–17]. In [15], the authors prove quadratic decay bounds and non-degeneracy at free boundary points assuming that $\textbf{a}_{ij}$ and *f* are bounded measurable and uniformly positive, see Lemma 2.7. In particular the authors show that the free boundary has zero Lebesgue measure. In [15–17] the authors discuss the regularity of the free boundary, additionally assuming that $a_{ij}, f \in VMO$.

### Summary of Main Results

We assume that $\textbf{a}$ is symmetric and satisfies the following uniform ellipticity condition: There exists a constant $\Lambda \in [1,\infty )$ such that, for almost every $x \in \mathbb {R}^d$ and every $\xi \in \mathbb {R}^d$,

1.13 $$\begin{aligned} \textbf{a}(x) \xi \cdot \xi \ge | \xi |^2 \quad \text{ and } \quad |\textbf{a}(x)| \le \Lambda \,. \end{aligned}$$

Our goal is to give a rather general condition on the coefficient matrix $\textbf{a}$, which allows to treat both *qualitative* and *quantitative* homogenization at the same time. Indeed, we show that under a suitable *sublinearity condition* for the correctors and the flux correctors, described in Sect. 2.3, we obtain homogenization estimates. More precisely, for $m = \lceil - \log _3 \varepsilon \rceil$, $\square _m := ( -\tfrac{1}{2} 3^m , \tfrac{1}{2} 3^m )^d$ and $e \in \mathbb {R}^d$, we let $\phi _{m,e} \in H^1(\square _m)$ solve the equation

1.14 $$\begin{aligned} \nabla \cdot \textbf{a}( e+ \nabla \phi _{m,e}) = 0 \quad \text{ in } \square _m, \end{aligned}$$

and consequently find a $3^m \mathbb {Z}^d$-periodic skew-symmetric ($\textbf{S}_{ij} = -\textbf{S}_{ji}$) tensor $\textbf{S}$ solving

1.15 $$\begin{aligned} \Delta \textbf{S}_{m,ij}^{e} = \partial _{x_j} \big ( \textbf{a}( e+ \nabla \phi _{m,e})\big )_i - \partial _{x_i} \big ( \textbf{a}( e+ \nabla \phi _{m,e})\big )_j, \quad \textbf{S}_{m,ij}^{e} \in H_{\textrm{per}}^1(\square _m) \,. \end{aligned}$$

The coarsened homogenized coefficients are defined via1.16Our assumption then reads as though there exists a homogenized matrix  and a monotone increasing function $t\mapsto \mathcal {E}(t)$ with $\mathcal {E}(0)$ such that1.17Our homogenization estimates are all given in terms of $\mathcal {E}(\varepsilon )$. Notice that $\mathcal {E}(\varepsilon )$ is centered at the origin and it is in fact a function of the coefficient field $\textbf{a}$ in $\square _m$. We suppress this dependence from the notation. However, one should keep in mind that $\mathcal {E}(\varepsilon ) = \mathcal {E}(\varepsilon , \textbf{a}|_{\square _m} )$ and we can thus extend the definition of $\mathcal {E}(\varepsilon )$ for any given point $x \in \mathbb {R}^d$ using translation by setting

1.18 $$\begin{aligned} \mathcal {E}(\varepsilon ,x) = \mathcal {E}(\varepsilon , \textbf{a}(x+\cdot ) |_{\square _m} ). \end{aligned}$$

For example, in the random case a typical form would be that $\mathcal {E}(\varepsilon ,x) = \mathcal {X}(x) \varepsilon ^\alpha$ for a stationary random field $x \mapsto \mathcal {X}(x)$ with certain stochastic integrability. Our goal in this paper, in a nutshell, is to show that *smallness of* $\mathcal {E}(\varepsilon )$ *implies large-scale regularity in the obstacle problem.* We would like to point out here, however, that we are not aiming at *optimal rates* of homogenization in the obstacle problem, since this would call much more careful analysis. We will not discuss this point any further in this manuscript.

For the regularity results, we need a stronger, more quantified control of the error. Indeed, for a given parameter $\nu \in (0,1)$, we assume that, for every $s,t \in (0,\infty )$ such that $s \le t$, we have that

1.19 $$\begin{aligned} s^{-\nu } \mathcal {E} (s) \le t^{-\nu } \mathcal {E}(t) \, . \end{aligned}$$

In particular, this is satisfied with $\mathcal {E}(\varepsilon ) = \mathcal {X}\varepsilon ^\nu$, where $\mathcal {X}$ is a universal constant possibly depending on the realization of the coefficient field $\textbf{a}$. Under the assumption (1.19), we also define the so called *minimal scale*: for every $\alpha ,\sigma \in (0,1]$ and $x \in \mathbb {R}^d$, set

1.20 $$\begin{aligned} R_{\sigma ,\alpha }(x) := \inf \bigl \{ r \in (0,\infty ) \, : \, \bigl ( \mathcal {E}\bigl ( \tfrac{\varepsilon }{r} , x \bigr )\bigr )^\alpha < \sigma \bigr \}, \qquad R_{\sigma ,\alpha } = R_{\sigma ,\alpha }(0) \,. \end{aligned}$$

This object is fundamental in the regularity theory, especially in the context of the stochastic homogenization, originating from the paper of Armstrong and Smart [9] in the random setting, see also [7, 32, 35]. The original ideas on large-scale regularity go back to seminal papers of Avellaneda-Lin [10–12].

Now let us introduce the results obtained in the current manuscript. We investigate the closeness of $u^\varepsilon$ and *u*, the corresponding free boundaries $\varGamma (u^\varepsilon )$ and $\varGamma (u)$, and derive large-scale regularity results for $u^\varepsilon$ and $\varGamma (u^\varepsilon )$.

The first theorem of the manuscript is the following:

#### Theorem 1.1

Let *U* be a bounded Lipschitz domain in $\square _0$. Let $\delta \in (0,\infty ]$. There exist constants $C(U,\delta ,d,\Lambda )<\infty$ and $\alpha (\delta ,d,\Lambda ) \in (0,\tfrac{1}{2})$ such that the following statement is valid. Suppose that $g \in W^{1,2+\delta }(U)$ and $f \in L^\infty (U)$ are such that $g \ge 0$ and $f>0$ almost everywhere in *U*. Let $u^\varepsilon \ge 0$ and $u \ge 0$ solve (1.1) and (1.7) respectively, with boundary values $u^\varepsilon =u=g$ on $\partial U$. Then we have the estimate1.21

Theorem 1.1 is the main homogenization result for the solutions to the obstacle problem, and it is essential in proving large-scale regularity results for the solution of the heterogenous obstacle problem and its free boundary.

Following [7], we denote by $\overline{\mathcal {A}}_k$ and ${\mathcal {A}}_k$ respectively the spaces of -harmonic and $\textbf{a}$-harmonic functions, with growth strictly less than polynomials of degree $k+1$. More rigorously,1.22and

1.23 $$\begin{aligned} {\mathcal {A}}_k := \bigl \{ u \in H^1_{loc}( \mathbb {R}^d) \, : \, -\nabla \cdot (\textbf{a}\nabla u )=0, ~ \lim _{r\rightarrow \infty } r^{-k-1} \Vert u \Vert _{{\underline{L}^2(B_{r})}}=0 \bigr \}. \end{aligned}$$

By the Liouville theorem, $\overline{\mathcal {A}}_1$ is the space of affine functions. Under the assumption (1.19), it turns out that ${\mathcal {A}}_1$ can be identified with the limits of $\phi _{m,e}$ in (1.14) as $m \rightarrow \infty$. The dimension of ${\mathcal {A}}_1$ is $d+1$ and it can be thought as a space of “heterogenous affine functions". This space gives the right concept to measure $C^{1,1}$-regularity of solutions of the obstacle problem. These type of large-scale regularity results were originally developed in the periodic setting by Avellaneda and Lin in a series of papers [10–12]. As a matter of fact, a version of the following theorem has been commented in the periodic non-divergence case already in [11]. Our contribution here is to prove the corresponding result also in the stochastic setting.

#### Theorem 1.2

Let $\nu \in (0,1]$ and assume that $\mathcal {E}$ satisfies (1.19). There exist constants $\alpha ,\sigma \in (0,1]$ and $C< \infty$, all depending only on $(\nu ,d,\Lambda )$, such that the following claim holds. If $R_{\sigma ,\alpha }$ defined in (1.20) satisfies $R_{\sigma ,\alpha }\le 1$, $f \in C^{0,\beta }(B_{1})$ and $u^\varepsilon$ is a weak solution of (1.1) in $B_1$, then there exists $\psi \in \mathcal {A}_1$ such that, for every $r\in [ R_{\sigma ,\alpha },1]$, we have

1.24 $$\begin{aligned} \Vert u^\varepsilon - \psi ^\varepsilon \Vert _{L^\infty (B_{r})} \le Cr^2 \big (\Vert u^\varepsilon \Vert _{\underline{L}^2(B_{1})} + C \Vert f \Vert _{C^{0, \nu }(B_1)} \big ) \,. \end{aligned}$$

Now let us discuss our results on the regularity of the free boundary $\varGamma (u^\varepsilon )$. Let $\lambda >0$, and define the set of homogeneous half-space solutions to the homogenized obstacle problem , as follows:1.25The following theorem is the second main result of this article:

#### Theorem 1.3

Let $\varepsilon \in (0,1)$, $\beta ,\vartheta \in (0,\tfrac{1}{2})$ and $\lambda > 0$. Let $\nu \in (0,1)$ and assume that the condition (1.19) is valid. There exist constants $\alpha (d,\Lambda )\in (0,1)$, $C(\nu ,\beta ,\vartheta ,\lambda ,d,\Lambda ) < \infty$, and $\sigma , r_0\in (0,1)$, $\gamma \in (0,r_0]$, all depending on $(\beta ,\vartheta ,\lambda ,d,\Lambda )$, such that the following claim is valid. Let $u^\varepsilon$ solve the normalized obstacle problem

1.26 $$\begin{aligned} \nabla \cdot (\textbf{a}^\varepsilon \nabla u^\varepsilon )= \lambda \chi _{\{ u^\varepsilon >0\} } \quad \text{ in } B_1, \end{aligned}$$

and assume that $0 \in \varGamma (u^\varepsilon )$. Suppose that

1.27 $$\begin{aligned} \inf _{r \in [\gamma r_0,\gamma ^{-1} r_0]} \frac{| \varLambda (u^\varepsilon ) \cap B_r|}{|B_r|} \ge \vartheta >0 \end{aligned}$$

and that $R_{\sigma ,\alpha } \le \gamma r_0$, where $R_{\sigma ,\alpha }$ is defined in (1.20). Then, for every $r \in [R_{\sigma ,\alpha },\frac{1}{2})$, there exists $h^{(r)} \in \mathbb {H}_\lambda ^0$ such that, for every $s \in [r,\frac{1}{2})$,

1.28 $$\begin{aligned} \Vert u^\varepsilon - h^{(r)} \Vert _{L^\infty (B_s)} \le C s^2 \Bigr ( s^{\beta } \inf _{h \in \mathbb {H}_\lambda ^0}\Vert u^\varepsilon -h \Vert _{L^2(B_{1})} + \bigl ( \mathcal {E}(\tfrac{\varepsilon }{r}) \bigr )^\alpha \Bigl ) \, . \end{aligned}$$

Motivated by the result of Theorem 1.3, we may give a definition of a regular free boundary points.

#### Definition 1.4

*(Large-scale regular free boundary point)* Let $\lambda >0$ and let $\alpha ,\sigma ,r_0,\gamma$ be from Theorem 1.3. Let $u^\varepsilon$ solve

1.29 $$\begin{aligned} \nabla \cdot (\textbf{a}^\varepsilon \nabla u^\varepsilon )= \lambda \chi _{\{ u^\varepsilon >0\} } \quad \text{ in } B_2 \,. \end{aligned}$$

We say that $x_0 \in \varGamma ( u^\varepsilon ) \cap B_1$ is a *large-scale regular free boundary point* if

1.30 $$\begin{aligned} \inf _{s \in [\gamma r,\gamma ^{-1} r]} \frac{| \varLambda (u^\varepsilon ) \cap B_s(x_0)|}{|B_s(x_0)|} \ge \vartheta >0 \quad \text{ and } \quad r \in \big [ \gamma ^{-1} R_{\sigma ,\alpha }(x_0) , r_0\big ] \,. \end{aligned}$$

A few comments are appropriate. First, the heart of the matter is to observe that all constants are independent of $\varepsilon$. This justifies the use of the word “quantified" in the title. Second, there is no regularity assumed from the matrix $\textbf{a}$ apart from the uniform ellipticity. The homogenization guarantees that $\mathcal {E}(\tfrac{\varepsilon }{r})$ can be taken to be small and that it is moreover *quantified*. In particular, in the periodic setting, $\mathcal {E}(t) = Ct$, so that by taking $r = C \delta ^{-1} \varepsilon$, we see that $\Vert u^\varepsilon - h \Vert _{L^\infty (B_r)} \le \delta r^2$ for every $r \in [C \delta ^{-1} \varepsilon , c \delta ^{1/\beta }]$. One could interpret this as “large-scale uniqueness of blow-ups" in the sense that the half space solution is fixed for all scales larger than $C\varepsilon$, which implies that also the normal direction of the free boundary is fixed in large-scale regularity sense. Similar types of statements remain valid in the stochastic case, but now the minimal scale $R_{\sigma ,\alpha }$ will be a *random object*.

Finally, there still remains a very interesting open problem, namely, under what condition one would be able to prove (or give a counterexample under some reasonable conditions) that the free boundary would be not only large-scale flat, but flat in the traditional sense. For example, as we were already discussing that in the periodic setting Theorem 1.3 yields large-scale regularity up to scales $C\varepsilon$. Assuming some smoothness of coefficients, Caffarelli’s results would imply regularity from $c\varepsilon$ scale on. Thus, the missing range is $[c\varepsilon ,C\varepsilon ]$. In fact, we suggest the following open problem for future investigation. Assume that the matrix $\textbf{a}$ is periodic and sufficiently regular. Can we then obtain uniform regularity results for the free boundary $\varGamma (u^\varepsilon )$, in a neighborhood of a regular free boundary point? In this setting, there exist blow-up solutions with respect to $u^\varepsilon$: for a fixed $x_0 \in \varGamma (u^\varepsilon )$ and a subsequence $r_j \rightarrow 0+$ there exists $u^\varepsilon _0(x):=\lim _{r_j \rightarrow 0+} \frac{u^\varepsilon (r_jx+x_0)}{r_j^2}$, and $\nabla \cdot \textbf{a}^\varepsilon (x_0) \nabla u^\varepsilon _0=\lambda \chi _{\{u_0^\varepsilon >0\}}$ in $\mathbb {R}^d$. However, the blow-ups of $u^\varepsilon$ depend on $\varepsilon$, and a very careful analysis with respect to both heterogenous and homogeneous halfspace blow-up solutions will be needed.

### Organization of the Manuscript

In Sect. 2 we gather the known homogenization estimates for the Dirichlet problem, and the known regularity theory for the obstacle problem. An expert in the field may skip this section. In Sect. 3 we obtain a quantitative homogenization result for the solution of the obstacle problem, employing the well-known penalization method for partial differential equations with discontinuous right hand side. The idea is the following: First we show the closeness of the solution to the obstacle problem to the penalized/regularized problem in the $$H^{1}$$-norm. Then we prove that a quantitative homogenization result holds for the regularized problem by following the two-scale expansion argument, and employing the known estimates for first order correctors. In Sect. 4 we prove large-scale $$ C^{1,1}$$- regularity for the solution of the obstacle problem with measurable coefficients. The Sect. 5 is devoted to the large-scale regularity of the free boundary. In the appendix we give two one-dimensional examples, with numerical illustrations, which further justify our assumptions.

## Background Theory

In this section we gather the known theory of homogenization and of the obstacle problem. It is done for the readers convenience, and an expert in either field can skip this section.

### Notations

Throughout the manuscript $$ \mathbb {R}^d$$ is the *d*-dimensional Euclidean space and the scalar product is denoted by $$ x\cdot y$$, for $$ x, y \in \mathbb {R}^d$$, $$ | \cdot |$$ is the Euclidean norm. The canonical basis is denoted by $$ \{ e_1,\ldots ,e_d \}$$. The ball with a centre at $$ x_0$$ and radius $$ r >0$$ is denoted by $$B_r(x_0)$$, when $$ x_0=0$$, we simply write $$B_r$$, and $$ B:=B_1$$. We denote, for $$m \in \mathbb {Z}$$, by $$\square _m$$ the triadic cube $$\square _m := (-\tfrac{1}{2} 3^m , \tfrac{1}{2} 3^m )^d$$.

Let $U \subseteq \mathbb {R}^d$. For a scalar valued function $f : U \rightarrow \mathbb {R}$, we denote the partial derivatives by $\partial _{x_i} f$. For a vector field $F: U \rightarrow \mathbb {R}^d$, we denote the divergence operator

$$\begin{aligned} \nabla \cdot F:= \sum _{i=1}^d \partial _{x_i} f_i, \end{aligned}$$

where $f_i = F \cdot e_i$. The Laplacian of a function *f* is denoted by

$$\begin{aligned} \Delta f= \nabla \cdot (\nabla f )=\sum _{i=1}^d \partial _{x_i} ( \partial _{x_i } f). \end{aligned}$$

By $\nabla ^k$ we denote the tensor of the *k*-th order partial derivatives of *f*.

#### Function Spaces

For a natural number $$ k \in \mathbb {N}$$, and $$ U \subseteq \mathbb {R}^d$$ the set of *k*-times continuously differentiable functions is denoted by $$ C^k$$. For $$ \alpha \in (0,1]$$ the Hölder seminorm is the following

$$\begin{aligned} {[} f ]_{ C^{0,\alpha }(U)}:= \sup _{x, y \in U, x \ne y} \frac{ | f(x)-f(y) |}{ | x-y|^\alpha }, \end{aligned}$$

and $C^{k, \alpha (U) }$ is the Hölder space, with the norm

$$\begin{aligned} \Vert f \Vert _{ C^{k, \alpha } (U)} :=\sum _{l =0}^k \sup _{ x\in U} | \nabla ^l f (x)| + [ \nabla ^k f ]_{ C^{0,\alpha }(U)}. \end{aligned}$$

For $$ p \in [1, +\infty ]$$, $$L^p(U) $$ is the Lebesgue space with the norm

$$\begin{aligned} \Vert f \Vert _{ L^p(U) } :=\left( \int _U |f(x)|^p \textrm{d}x \right) ^\frac{1}{p}, \text { for } p< +\infty , \text { and } \Vert f \Vert _{ L^\infty (U) } := {\mathop {\mathrm{ess\,sup}}\limits _{x \in U}}\, |f(x)|. \end{aligned}$$

If $$ | U| <+\infty $$ (*U* has a finite Lebesgue measure) thenthe average of *f* on *U*. We will often use the averaged norms:The standard notation $$ W^{k,p} (U )$$ for Sobolev spaces is used, with the norm

$$\begin{aligned} \Vert f \Vert _{ {W}^{k,p}(U) }:= \sum _{l=0}^k \Vert \nabla ^l f \Vert _{ {L}^p(U) }, \end{aligned}$$

and the averaged norm is defined as follows:

$$\begin{aligned} \Vert f \Vert _{ \underline{W}^{k,p}(U) }:= \sum _{l=0}^k |U |^{\frac{l-k}{d} } \Vert \nabla ^l f \Vert _{ \underline{L}^p(U) }. \end{aligned}$$

The notation $$ W_0^{k,p} (U )$$ is used for Sobolev spaces with zero trace. We also use the notation $$H^k = W^{k,2}$$ for every $$k \in \mathbb {N}$$. The dual space of $$H_0^1(U)$$ is denoted by $$H^{-1}(U)$$.

### Useful Inequalities

We will also make use of the following standard interpolation result many time (for the sketch of the proof, see [7, Exercise 3.20]):

#### Lemma 2.1

Let $\alpha \in (0,1]$ and $r \in (0,\infty )$. Then there exists a constant $C(\alpha ,d)<\infty$ such that, for every $u\in C^{0,\alpha }(B_r)$, we have

$$\begin{aligned} \Vert u \Vert _{L^{\infty }(B_r)} \le C \Vert u \Vert _{\underline{L}^{2}(B_r)}^{\frac{2\alpha }{d+2\alpha }} \big ( r^\alpha [ u ]_{C^{0,\alpha }(B_r)} \big )^{\frac{d}{d+2\alpha }} . \end{aligned}$$

The following classical result can be found, for example, in [36]:

#### Lemma 2.2

(Meyers lemma) Fix $$ r >0$$ and $$ p \in (2, \infty )$$. Suppose that $$ f \in W^{-1,p}$$ and that $$ u \in H^1(B_r)$$ satisfy

2.1 $$\begin{aligned} -\nabla \cdot ( \textbf{a}\nabla u) =f \text { in } B_r. \end{aligned}$$

Then there exists $\delta (p,d, \Lambda ) > 0$ and $C (p,d, \Lambda ) <\infty$ such that

$$\begin{aligned} \Vert \nabla u \Vert _{ \underline{L}^{ (2+\delta ) \wedge p} (B_{r/2})} \le C \left( \Vert \nabla u \Vert _{ \underline{L}^{ 2} (B_{r})} + \Vert f \Vert _{ \underline{W}^{ -1, (2+\delta ) \wedge p} (B_{r})} \right) \end{aligned}$$

Furthermore, the following global version holds: if *u* solves

2.2 $$\begin{aligned} -\nabla \cdot ( \textbf{a}\nabla u) =f \text { in } U, \end{aligned}$$

where *U* is a Lipschitz domain, and $u \in g +H^{1}_0(U)$, with $g \in W^{1,p}(U)$,then

$$\begin{aligned} \Vert \nabla u \Vert _{ \underline{L}^{ (2+\delta ) \wedge p} (U)} \le C \left( \Vert \nabla g \Vert _{ \underline{L}^{ 2} (U)} + \Vert f \Vert _{ \underline{W}^{ -1, (2+\delta ) \wedge p} (U)} \right) \end{aligned}$$

We shall often refer to the following De Giorgi-Nash-Moser theorem:

#### Theorem 2.3

(De Giorgi-Nash-Moser, Theorem 8.22 in [30]) Let $p >\tfrac{d}{2}$. Let *U* be an open set, $f\in L^p(U)$ and let $u \in H^{1}(U)$ solve

$$\begin{aligned} \nabla \cdot (\textbf{a}\nabla u)= f\quad \text {in } U, \end{aligned}$$

where $\textbf{a}$ satisfies the uniform ellipticity assumption (1.13). Then *u* is locally a Hölder continuous function in *U*. In particular, there exist constants $C(p, r_0,d, \Lambda ) \in [1,\infty )$ and $\alpha =\alpha (p,d,\Lambda ) \in (0,1)$ such that if $B_{2r}(x) \subseteq U$ for some $r>0$ and $x\in U$, then, for every $t \in (0,r)$,

2.3 $$\begin{aligned} \Vert u \Vert _{L^\infty (B_r(x)) } + \Bigl ( \frac{r}{t} \Bigr )^\alpha \Vert u - u(x) \Vert _{L^\infty (B_t(x)) } \le C \left( \Vert u \Vert _{\underline{L}^2(B_{2r}(x)) } + r^2 \Vert f \Vert _{\underline{L}^p(B_{2r}(x))}\right) .\nonumber \\ \end{aligned}$$

### Homogenization Theory, Assumptions and Known Estimates

Before proceeding to the discussion of our problem and main results obtained in the manuscript, let us give some basic notations, and a short discussion of key ideas and concepts in the homogenization theory. A very thorough discussion on many aspects of homogenization as well as rather comprehensive bibliographical references can be found in the monograph of Jikov, Kozlov & Oleĭnik [37].

#### Periodic Homogenization

Possibly the most studied branch of homogenization, so far, is the case of deterministic, periodic coefficients, that is, $\textbf{a}(x + z) = \textbf{a}(x)$ for every $z \in \mathbb {Z}^d$ and for almost every $x \in \mathbb {R}^d$. Higher dimensional works start from the works of De Giorgi and Spagnolo [23] using $\Gamma$-convergence, followed by direct methods of Tartar, see [46] and the references therein, and two-scale analysis of Bensoussan et al. [13]. For the contemporary analysis we refer to an excellent monograph of Shen [45]. In the periodic case, we find $\mathbb {Z}^d$-periodic correctors $\phi _{e_k}$ and $\textbf{S}^{(e_k)}$ solving (1.14) and (1.15) with $m = 0$, respectively, so that $\phi _{e_k,m}$ and $\textbf{S}_m^{(e_k)}$ are simply periodic extensions of these. In particular, there exists a constant $C(d,\Lambda ) <\infty$ such that, for every $\varepsilon \in (0,1]$,

2.4 $$\begin{aligned} \mathcal {E}(\varepsilon ) := C \varepsilon . \end{aligned}$$

Homogenized matrix can be defined using the flux of the corrector, that is, for every $e \in \mathbb {R}^d$,2.5and we may set .

#### Almost Periodic Homogenization

Homogenization of elliptic equations with almost-periodic coefficients was studied first by Kozlov [38]. The quantified theory was initialized in [44] and later on with large-scale regularity and more quantified rates in [8]. The necessary condition for algebraic rate (1.19) is that there exists a constant $\beta \in (0,1)$ such that, for $R \gg 1$,

$$\begin{aligned} \sup _{y \in \mathbb {R}^d} \inf _{z \in B_R } \Vert \textbf{a}(\cdot +y) - \textbf{a}(\cdot +z)\Vert _{L^\infty (\mathbb {R}^d)} \le R^{-\beta } \end{aligned}$$

Under stronger quantitative assumptions on $\textbf{a}$ it is also possible to prove (2.4), see [3], and now $\mathcal {E}(\varepsilon ) := C \varepsilon ^{\alpha }$ for some small $\alpha (d,\beta ) \in (0,1)$.

#### Stochastic Homogenization

Stochastic homogenization has been under intensive research for the last 10–15 years. Early contributions in qualitative theory were made already earlier, in 1980s, by Yuriinski [47], Kozlov [39] and Papanicolaou and Varadhan [41]. Let us also mention here the variational methods developed by Dal Maso and Modica [21, 22]. The starting point for the quantitative theory, which is also the backbone of this paper, was the unpublished manuscript of Naddaf and Spencer. The main tools in this paper were concentration inequalities, such as the Efron-Stein or logarithmic Sobolev inequalities. These ideas were pushed forward by Gloria and Otto, starting from [34]. They, together with Fischer and Neukamm and many others [26, 32], developed the theory of the quantitative homogenization, see references in [33]. Later, a different approach was taken by Armstrong and Smart [9], pushing much further the variational approach of Dal Maso and Modica. The important contribution of this paper was that it developed the large-scale regularity following Avellaneda and Lin in the context of stochastic homogenization. Moreover, these techniques were developed further by Armstrong and Mourrat [5, 6] and the second author of this paper, and much of the theory is summarized in the monograph [7]. See also [35].

Let $U \subseteq {\mathbb {R}^d}$ be a Lipschitz domain, and $p\subseteq {\mathbb {R}^d}$, and $\ell _p(x):=p\cdot x$. Denote by2.6and by $v(\cdot , U,p)$ the unique minimizer of this energy. Then

$$\begin{aligned} -\nabla \cdot ( \textbf{a}\nabla v(\cdot ,U,p))=0 \text { in } U, \quad v = \ell _p \text { on } \partial U. \end{aligned}$$

It is easy to show that the mapping $p\mapsto \mu (U,p)$ is a positive quadratic form and subadditive with respect to partitions of *U*. We denote by $\textbf{a}_m$ the matrix having components2.7Under some mild ergodicity assumption, the limit  exists almost surely. The matrix  satisfies the same ellipticity conditions as $\textbf{a}$, and it is called the homogenized matrix.

For every $m \in \mathbb {N}$ and $e \in \mathbb {R}^d$, we set

2.8 $$\begin{aligned} \phi _{m,e}(x):= v(x,\square _m,e)-e\cdot x. \end{aligned}$$

Observe that $\phi _{m,e}$ satisfies the Eq. (1.14) in $\square _m$. Having defined the finite volume corrector $\phi _{m,e}$ and extending it periodically to the whole $\mathbb {R}^d$, we define the finite volume flux corrector as a $3^m \mathbb {Z}^d$-periodic function $\textbf{S}_m^{e}$ such that its components are, for every $i,j \in \{1,\ldots ,d\}$, unique solutions of the equations

2.9 $$\begin{aligned} \Delta \textbf{S}_{m,ij}^{e} = \partial _{x_j} \big ( \textbf{a}( e+ \nabla \phi _{m,e})\big )_i - \partial _{x_i} \big ( \textbf{a}( e+ \nabla \phi _{m,e})\big )_j, \quad \textbf{S}_{m,ij}^{e} \in H_{\textrm{per}}^1(\square _m) \end{aligned}$$

Indeed, we see by taking divergence that $\sum _{j=1}^d \partial _{x_j} \textbf{S}_{m,ij}^{e} - \big ( \textbf{a}( e+ \nabla \phi _{m,e})\big )_i$ is a periodic harmonic function in $\mathbb {R}^d$, and as such it is a constant. Since the equation is invariant under constant addition, we may take that constant to be . Moreover, we have the following estimate for $\textbf{S}_{m,ij}^{e}$, for every $i,j \in \{1,\ldots ,d\}$ and $e \in B_1$,2.10where the weak norm on the right is defined asWe next give an example of assumptions in stochastic homogenization, namely the case of finite range dependence. Endow $\Omega$, the set of symmetric matrices with eigenvalues on the interval $[1,\Lambda ]$, with a family of $\sigma$-algebras $\left\{ \mathcal {F}_U \right\}$ indexed by the family of Borel subsets $U\subseteq {\mathbb {R}^d}$, defined by

2.11 $$\begin{aligned} \mathcal {F}_U:= &   \text{ the } ~\sigma \text{-algebra } \text{ generated } \text{ by } \text{ the } \text{ following } \text{ family: } \nonumber \\  &   \left\{ \textbf{a}\mapsto \int _{U} \textbf{a}_{ij} (x) \varphi (x)\,dx \, : \, \varphi \in C^\infty _c(U), \ i,j\in \{1,\ldots ,d\} \right\} . \end{aligned}$$

The largest of these $\sigma$-algebras is denoted $\mathcal {F}:= \mathcal {F}_{{\mathbb {R}^d}}$. For each $y\in {\mathbb {R}^d}$, we let $T_y:\Omega \rightarrow \Omega$ be the action of translation by *y*,

2.12 $$\begin{aligned} \left( T_y\textbf{a}\right) (x):= \textbf{a}(x+y), \end{aligned}$$

and extend this to elements of $\mathcal {F}$ by defining $T_y E:= \left\{ T_y\textbf{a}\,:\, \textbf{a}\in E \right\}$. Assume that the probability measure $\mathbb {P}$ on the measurable space $(\Omega ,\mathcal {F})$ satisfy the following two assumptions.

- *Stationarity with respect to* $\mathbb {Z}^d$-*translations:*
  2.13 $$\begin{aligned} \mathbb {P}\circ T_z = \mathbb {P}\quad \text{ for } \text{ every } \ z\in \mathbb {Z}^d. \end{aligned}$$
- *Unit range of dependence*.
  2.14 $$\begin{aligned}  &   \mathcal {F}_U \ \text{ and } \ \mathcal {F}_V \ \ \text{ are } ~\mathbb {P}\text{-independent } \text{ for } \text{ every } \text{ pair }~U,V\subseteq {\mathbb {R}^d}\nonumber \\  &   \quad \text{ of } \text{ Borel } \text{ subsets } \text{ satisfying }~{{\,\textrm{dist}\,}}(U,V)\ge 1. \end{aligned}$$

We denote the expectation with respect to $\mathbb {P}$ by $\mathbb {E}$. That is, if $\mathcal {X}:\Omega \rightarrow \mathbb {R}$ is an $\mathcal {F}$-measurable random variable, we write

2.15 $$\begin{aligned} \mathbb {E}\left[ \mathcal {X}\right] := \int _\Omega \mathcal {X}(\textbf{a}) \, \textrm{d}\mathbb {P}(\textbf{a}). \end{aligned}$$

While most of the objects in our study are functions of $\textbf{a}\in \Omega$, we do not typically display this dependence explicitly in our notations. For a random variable $\mathcal {X}$ and parameters $s, \theta \in (0, \infty )$, we use the abbreviation

2.16 $$\begin{aligned} \mathcal {X}\le \mathcal {O}_s(\theta ) \qquad \Longleftrightarrow \qquad \mathbb {E}\left[ \exp (\theta ^{-1} \mathcal {X}_+)^s \right] \le 2 . \end{aligned}$$

Now, following [4, 7], one can show that under these assumptions, there is a constant $C(d,\Lambda )$, a random variable $\mathcal {X}$ satisfying $\mathcal {X}\le \mathcal {O}_d(C)$ and an exponent $\gamma (d,\Lambda ) \in (0,1]$ such that

2.17 $$\begin{aligned} \mathcal {E}(\varepsilon ) := ( \mathcal {X}\varepsilon )^\gamma . \end{aligned}$$

There are of course other mixing conditions allowing the above estimate for the sublinearity of the correctors, starting from [35]. Much more in general, there is a general unified condition, a *concentration for sums*, that allows to treat most of the well-known cases from the literature. Indeed, in [4] one can find numerous examples leading to the condition (2.17), with the stochastic integrability stemming to the particular model.

We will conclude the discussion about homogenization with an important remark. In the random setting, $\mathcal {E}(\varepsilon )$ itself is a random object. Under stationarity assumptions, it has its stationary extension. Notice that our results are all centered at the origin. When considering the translations of these results, which are of course trivial in the case of Laplacian or periodic coefficients, one has to be careful in the random setting; the minimal scale and $\mathcal {E}(\varepsilon )$ will change, in general, from point to point, see (1.18).

### The Classical Obstacle Problem

First we give a brief summary of the classical obstacle problem, following Caffarelli [18, 20]. The main purpose here is to describe the main tools and techniques in investigation of the regularity of the free boundary.

As before $U \subseteq \mathbb {R}^d$ is a given Lipschitz domain and $\varphi \in H^{1}(U)$ is the obstacle. Then the minimizer to the following functional

2.18 $$\begin{aligned} J[u]= \int _\Omega | \nabla u(x) |^2 \textrm{d}x, \end{aligned}$$

over all functions $u=g~$, $\varphi < g$ on $\partial U$, such that $u\ge \varphi$ in *U*, is called the solution to the obstacle problem with obstacle $\varphi$. The solution satisfies the following variational inequalities

2.19 $$\begin{aligned} \Delta u\le 0, ~ u \ge \varphi , ~ \Delta u \cdot (u-\varphi )=0. \end{aligned}$$

The following set $\varOmega (u):= U \cap \{ u>\varphi \}$ is called the non-coincidence set, and $\varGamma (u):=\partial \Omega (u) \cap U$ is called the free boundary.

It is well known that the solution to the obstacle problem is as regular as the obstacle up to $C^{1,1}$, [28].The solution *u* cannot obtain a better regularity, since $\Delta u$ is discontinuous on $\varGamma (u)$. Let $v:= u-\varphi \ge 0$, then $\Delta v =- \Delta \varphi \chi _{ \{ v >0 \}}$. Denoting by $f:=- \Delta \varphi \ge 0$, we derive the normalized obstacle problem

2.20 $$\begin{aligned} \Delta v =f \chi _{ \{ v >0 \}}. \end{aligned}$$

It is clear that the properties of the normalized obstacle problem above can be easily adapted for the following obstacle problem, , with the homogenized matrix .

#### Theorem 2.4

(Frehse [28]) Let $\overline{u} \ge 0$ be the solution to the obstacle problem  in a domain *U*. Assume that $B_{2r}(z) \subseteq U$ and there exists a $\psi \in C^{1,1}$, such that , then

2.21 $$\begin{aligned} \Vert D^2 {\overline{u}} \Vert _{L^\infty (B_{r})} \le C(d, \Lambda ) \left( \frac{\Vert {\overline{u}} \Vert _{L^\infty (B_{2r})}}{r^2} + \Vert D^2 \psi \Vert _{L^\infty (B_{2r})}\right) , \end{aligned}$$

In particular, if $f \in C^{0,\beta }(B_{2r}(z))$, then there exists a constant $C(\beta ,d,\Lambda )< \infty$ such that

2.22 $$\begin{aligned} \Vert D^2 {\overline{u}} \Vert _{L^\infty (B_{r})} \le C(\beta , d, \Lambda ) \left( \frac{\Vert {\overline{u}} \Vert _{L^\infty (B_{2r})}}{r^2} +\Vert f \Vert _{L^\infty (B_{2r})} + r^\beta [ f ]_{C^{0, \beta }(B_{2r})}\right) . \end{aligned}$$

The proof of (2.21) can be easily found, for instance in [42], and (2.22) follows by the Schauder estimates.

The regularity of the free boundary $\varGamma (u)$ has been well studied in the literature [18, 20]. It is customary to take $f \equiv 1$, since the more general case $f \ge c>0$, under regularity assumptions on *f*, can be investigated following similar techniques, [14]. Fix $x_0 \in \varGamma (u)$, then the solution has a quadratic decay at $x_0$

2.23 $$\begin{aligned} \sup _{B_r(x_0)} u\le C r^2. \end{aligned}$$

On the other hand for any $x_0 \in \overline{ \{ u>0 \} } \cap U$, and any $B_r(x_0) \subseteq U$,

2.24 $$\begin{aligned} \sup _{B_r(x_0)} u\ge c r^2, \end{aligned}$$

which is the non-degeneracy property.

Denote by

$$\begin{aligned} u_{r,x_0}:=\frac{u(x_0 +rx )}{r^2}, \end{aligned}$$

then $u_{r_j, x_0} \rightarrow u_0$ up to a subsequence $r_j \rightarrow 0+$. The limit function $u_0$ is a global solution to the obstacle problem $\Delta u_0=\chi _{\{ u_0>0\}}$, and is called a *blow-up solution*. We can understand the structure of the free boundary at $x_0$ by analyzing the blow-up limits at that point.

#### Theorem 2.5

(Caffarelli [18, 20]) Let $$ \overline{u}$$ be the solution to the obstacle problem , and $$ x_0$$ be a free boundary point. Then the blow-up of $$\overline{u}$$ at $$x_0$$, $$u_0$$, is unique, and there are two possibilities. Either $$u_0$$ is a half-space solution, that is,2.25and $$x_0$$ is a called a regular free boundary point. In a neighborhood of each regular free boundary point $$\varGamma (u)$$ is an analytic surface. Otherwise, $$u_0$$ is a second order nonnegative polynomial,2.26In this case, $$x_0$$ is called a singular free boundary point, and all such points are lying on a lower dimensional $$C^1$$-manifold.

#### The Divergence form Obstacle Problem with Measurable Coefficients

Now we consider the obstacle problem

2.27 $$\begin{aligned} \nabla \cdot (\textbf{b}\nabla u)= f \chi _{ \{ u>0\}}, \qquad f \ge \lambda > 0, \end{aligned}$$

where $\textbf{b}$ is an elliptic ($I_d \le \textbf{b}\le \Lambda I_d$) and symmetric matrix, with measurable coefficients, and *f* is a measurable function satisfying the lower bound in (2.27).

##### Lemma 2.6

Fix $q \in (\frac{d}{2},\infty )$ and $\delta \in (0,1)$. Suppose that $g \in W^{1,2+\delta }(U)$ and $f \in L^q(U)$ for $q \in (\frac{d}{2},\infty )$

- Global estimates. There exists constants $C(U,d,\Lambda )< \infty$ and $\alpha (\delta ,d,\Lambda )\in (0,1)$ such that
  2.28 $$\begin{aligned} \left\| \nabla u^\varepsilon \right\| _{L^{2+\alpha } \left( U \right) } + \left\| \nabla u \right\| _{L^{2+\alpha } \left( U \right) } \le C \big ( \Vert \nabla g \Vert _{L^{2+\delta }(U)}+\Vert f\Vert _{L^{2+\delta }(U)} \big ). \end{aligned}$$
- Local estimates. There exists a constant $C(d,\Lambda )< \infty$ and $\alpha (d,\Lambda ) \in (0,1)$ such that if $B_{2r}(z) \subseteq U$, then
  2.29 $$\begin{aligned}  &   \left\| u^\varepsilon \right\| _{L^{\infty } \left( B_{r}(z) \right) } + r^\alpha \left[ u^\varepsilon \right] _{C^{0,\alpha } \left( B_{r}(z) \right) } + r \left\| \nabla u^\varepsilon \right\| _{L^{2+\alpha } \left( B_{r}(z) \right) } \nonumber \\  &   \quad \le C \big ( \left\| u^\varepsilon - (u^\varepsilon )_{B_{2r}(z)} \right\| _{\underline{L}^{2} \left( B_{2r}(z) \right) } + r^2 \Vert f\Vert _{\underline{L}^{q}(B_{2r}(z))} \big ) . \end{aligned}$$

Here (2.28) follows by treating $f \chi _{\{u^\varepsilon > 0\}}$ as bounded right hand side. The Hölder regularity estimate in the scaling invariant form is also classical, see [36].

Next we would like to state the properties of the obstacle with rough coefficients, following Ivan Blank and collaborators, [15–17].

##### Lemma 2.7

(Blank and Hao [15]) Let $I_d \le \textbf{a}\le \Lambda I_d$, be a symmetric matrix, with bounded measurable coefficients and $f \ge \lambda >0$ be a bounded measurable function. There exist constants  $0< c<C< \infty$, both depending only on the given data $(\lambda ,d,\Lambda )$, such that for any $u \ge 0$ solution of the divergence-form obstacle problem

2.30 $$\begin{aligned} \nabla \cdot (\textbf{a}\nabla u)=f \chi _{\{ u>0\}}, \text { in } U \end{aligned}$$

the following statements hold:

*Quadratic decay* ([15, Theorem 3.2]): If $x_0 \in \varGamma (u)$ is a fixed free boundary point, then

2.31 $$\begin{aligned} \sup _{B_r(x_0)} u \le C\Vert f \Vert _{L^\infty (U)} r^2. \end{aligned}$$

*Non-degeneracy* ([15, Theorem 3.9]): If $x_0\in \overline{\{ u >0 \} } \cap U$, then

2.32 $$\begin{aligned} \sup _{B_r(x_0)} u \ge c r^2, \end{aligned}$$

provided $$ B_{2r}(x_0)\subseteq U$$.

If $$ \textbf{a}=I_d$$, then in [14] Blank gives an example, showing that the blow-ups are not unique if *f* is merely continuous. If $$ a_{ij}$$ and *f* are merely bounded and measurable, we cannot even define the blow-up solutions, see Sect. 5. There is a counterexample in [15] showing that if $$\textbf{a}$$ is discontinuous then $$\lim _{r\rightarrow 0+} u_r$$ does not exists, even when imposing a uniform positive density assumption on the coincidence set $$ \{u \equiv 0\}$$ at free boundary points. In [16], see also [17], the authors show the existence of blow-ups assuming that $$ \textbf{a}_{ij}, f \in VMO$$, they also show that in general the blow-ups are not unique by constructing a counterexample.

## Homogenization of the Obstacle Problem

In this section we will prove the core homogenization result for the obstacle problem, Theorem 1.1. We will derive the homogenization result for the obstacle problem in two steps. First, we compare the solution of the obstacle problem with the solution of the penalized problem, for which merely measurable coefficients suffice. Second, we prove the actual homogenization estimates for the penalized problem, and after that tie these together simply by the triangle inequality.

We will prove several lemmas and propositions which combined eventually will lead to the homogeniztion for both the normalized and classical obstacle problems.

### Closeness of the Two Obstacle Problems

We claim that the homogenization of the solution to the normalized obstacle problem imply homogenization of the solution to the original obstacle problem, i.e. with given obstacle $\varphi \in H^1(U)$. For this, consider the problems3.1with $w\in W^{1,2+\beta } (U) \cap W_{\textrm{loc}}^{2,\infty } (U)$, $\beta \in (0,\infty ]$.

#### Lemma 3.1

Fix $\beta \in (0,1)$ and let $U \subseteq B_1$ be a Lipschitz domain. Then there exist constants $\delta (\beta ,d,\Lambda ) \in (0,\beta ]$ and $C(U,\beta ,d,\Lambda )< \infty$ such that the following holds. If $w^\varepsilon \in W^{1,2+\beta } (U)$ and $w \in W^{1,2+\beta } (U) \cap W_{\textrm{loc}}^{2,\infty } (U)$ solve (3.1), then, for every $r \in (0,1)$,

3.2 $$\begin{aligned} \Vert w^\varepsilon - w\Vert _{L^{2} (U)}&\le C \big (\mathcal {E}(\varepsilon ) + r^{\frac{\delta }{2+\delta } } \big ) \Vert \nabla w \Vert _{L^{2+\delta } (U)} \nonumber \\&\quad + C \mathcal {E}(\varepsilon ) \big ( r^{-1} \Vert \nabla w \Vert _{L^{\infty } (U_r)} + \Vert \nabla ^2 w \Vert _{L^{\infty } (U_r)} \big ) , \end{aligned}$$

where $$U_r = \{ x\in U \, : \, {{\,\textrm{dist}\,}}(x,\partial U) > r \}$$.

#### Proof

First, define the two-scale expansion of $\varphi$ as

$$\begin{aligned} \theta ^\varepsilon (x) = w(x) + \varepsilon \eta \sum _{k=1}^d \partial _{x_k} w(x) \phi _{e_k}\big (\tfrac{x}{\varepsilon } \big ) \end{aligned}$$

with $\eta \in C_0^\infty (U)$ being a cutoff function such that $\mathbb {1}_{U_r} \le \eta \le \mathbb {1}_{U}$. By a direct computation (see [7, Chapters 1 and 6] or the computation in the proof of Proposition 3.4)whereBy equations of $w^\varepsilon$ and *w*, the Poincaré inequality, and the fact that $w^\varepsilon = \theta ^\varepsilon$ on $\partial U$, we obtain that

$$\begin{aligned} \Vert w^\varepsilon - \theta ^\varepsilon \Vert _{H^{1}(U)} \le C \Vert \textbf{g}^\varepsilon \Vert _{L^{2}(U)} \,. \end{aligned}$$

Therefore, by the triangle inequality and (1.17), we arrive at

$$\begin{aligned}&\Vert w^\varepsilon - \theta ^\varepsilon \Vert _{H^{1}(U)} \le C \big (\mathcal {E}(\varepsilon ) {+} r^{\frac{\delta }{2+\delta } } \big ) \Vert \nabla w \Vert _{L^{2+\delta } (U)}\\&\quad + C \mathcal {E}(\varepsilon ) \big ( r^{-1} \Vert \nabla w \Vert _{L^{\infty } (U_r)} {+} \Vert \nabla ^2 w \Vert _{L^{\infty } (U_r)} \big ) . \end{aligned}$$

Applying then once more the bound (1.17) for $\phi _{e_k}$ completes the proof. $\square$

We also record the comparison principle for the solutions.

#### Lemma 3.2

(Comparison principle) Let *U* be a Lipschitz domain and $g\in H^1(U)$. Suppose that $\varphi$ and ${\widetilde{\varphi }}$ are two continuous obstacles in $H^1(U)$ and let *u* and ${\widetilde{u}}$ be solutions to the obstacle problem with boundary values *g* on $\partial U$ and continuous up to the boundary. Then

$$\begin{aligned} \Vert u - {\widetilde{u}} \Vert _{L^\infty (U)} \le \Vert \varphi - {\widetilde{\varphi }} \Vert _{L^\infty (U)} . \end{aligned}$$

#### Proof

Let $\delta = \Vert \varphi - {\widetilde{\varphi }} \Vert _{L^\infty (U)}$ and suppose that $\delta > 0$. Assume, on the contrary to the claim, that there is a point $x \in U$ such that $u(x) > {\widetilde{u}}(x) + \delta$. Then, by continuity, there is a domain *V* in the interior of *U* such that $u > {\widetilde{u}} + \delta$ in *V* and $u = {\widetilde{u}} + \delta$ on $\partial V$. Indeed, $u = {\widetilde{u}}$ on $\partial U$, and hence $\partial V$ cannot touch $\partial U$. Now, in *V* we have that *u* is above $\varphi$ and hence it is a weak solution in *V*. On the other hand, ${\widetilde{u}} + \delta$ is a weak supersolution in *V*, and since ${\widetilde{u}} + \delta = u$ on $\partial V$, the minimum principle implies that ${\widetilde{u}} + \delta \ge u$ in *V*; a contradiction. Thus the claim follows. $\square$

### Penalized Obstacle Problem

The penalization method is a well-known argument, which is widely used in the regularity theory of variational problems, see for example [29]. The core idea of the method is the following: Given a solution *u* to a partial differential equation with a discontinuous right hand side, we consider the solution of the corresponding partial differential equation with a regularized right hand side, with the same boundary values as the given function *u*. Showing that the given solution *u* is approximated by a more regular function, we then obtain regularity estimates for the given function *u*.

In this manuscript we use the penalization method in order to obtain quantitative homogenization for the obstacle problem. Below we describe the method for the obstacle problem, and derive the approximation lemma, even though we believe it can be found in the literature.

Let $U \subseteq \mathbb {R}^d$ be a given open bounded domain, with Lipschitz boundary, and $\textbf{b}$ be a given uniformly elliptic matrix, with measurable coefficients in *U*, satisfying the ellipticity condition $|\xi |^2 \le \textbf{b}(\cdot ) \xi \cdot \xi \le \Lambda |\xi |^2$. Let $f \in L^\infty (U)$ be a given bounded function satisfying $f >0$ a.e.. Denote by *u* the unique minimizer of the functional

3.3 $$\begin{aligned} J[v, \textbf{b}]=\int _U \frac{1}{2}\nabla v \cdot (\textbf{b}\nabla v ) +f v \, \textrm{d}x \end{aligned}$$

over $v \in W^{1,2}(U)$, $v \ge 0$, satisfying the boundary condition $v=g>0$ on $\partial U$. Then *u* satisfies the following variational inequality in a weak sense:

3.4 $$\begin{aligned} \nabla \cdot (\textbf{b}\nabla u )=f\chi _{\{u>0\}}, ~u \ge 0 \text { in } U,~ u=g \ge 0 \text { on } \partial U. \end{aligned}$$

We see that the right hand side in the partial differential equation in (3.4) is a discontinuous function. The penalization method is studying an approximating problem for (3.4), with regularized right hand side. For this, let $\beta :(-\infty , +\infty )\rightarrow [-1,1]$ be an approximation of the function $\chi _{\{v>0\}}$, for simplicity, we take  $\beta$ to be the Lipschitz continuous function

3.5 $$\begin{aligned} \beta (t)=t~ \text { for }~ |t| \le 1 \quad \text { and } \quad \beta ({t})= t/|t| ~ \text { for } ~ | t| > 1, \end{aligned}$$

and denote by $\gamma \in C^{1,1}$ its integral function $\gamma (t) := \int _0^t \beta (t') \, \textrm{d}t'$. Let

3.6 $$\begin{aligned} \beta _s(t):=\beta (\tfrac{t}{s}) \quad \text { and } \quad \gamma _s(t)=s\gamma (\tfrac{t}{s}) \quad \text { so that } \quad \gamma _s^\prime (t)= \beta _s(t) . \end{aligned}$$

Denote by $u^s$ the unique minimizer of the energy

3.7 $$\begin{aligned} J_s[v, \textbf{b}]=\int _U \tfrac{1}{2}\nabla v \cdot (\textbf{b}\nabla v ) +f\gamma _s(v)\textrm{d}x \end{aligned}$$

over $v \in H^{1}(U)$, satisfying the boundary condition $v=g \ge 0$ on $\partial U$, or, equivalently,

3.8 $$\begin{aligned} \nabla \cdot (\textbf{b}\nabla u^s) =f \beta _s( u^s), ~u^s =g \ge 0\text { on } \partial U. \end{aligned}$$

#### Lemma 3.3

Let *U* be a Lipschitz domain. Let $q \in ( \frac{d}{2}, \infty ]$ and let $f \in L^q(U)$ be a given function such that *f* is positive almost everywhere in *U* and let $g \in H^1(U)$ be a nonnegative function in *U*. Let $\textbf{b}$ be a symmetric matrix satisfying the ellipticity condition $|\xi |^2 \le \textbf{b}(\cdot ) \xi \cdot \xi \le \Lambda |\xi |^2$. If *u* is the solution to the obstacle problem

$$\begin{aligned} \nabla \cdot ( \textbf{b}\nabla u )=f\chi _{\{ u>0 \}}, \quad&u - g \in H_0^1(U) \end{aligned}$$

and $u^s$ solves the penalized equation

3.9 $$\begin{aligned} \nabla \cdot (\textbf{b}\nabla u^s) =f \beta _s( u^s), ~ u^s - g \in H_0^1(U), \end{aligned}$$

then $u^s$ is a nonnegative continuous functions and

3.10 $$\begin{aligned} 0 \le \nabla \cdot (\textbf{b}\nabla u^s) \le f \text { in a weak sense}. \end{aligned}$$

Furthermore, $u^s \rightarrow u$ as $s\rightarrow 0+$ with the following quantitative estimates:

3.11 $$\begin{aligned} 0 \le u^s-u\le s \quad \text {in } U \end{aligned}$$

and

3.12 $$\begin{aligned} \Vert \nabla u^s-\nabla u\Vert ^2_{L^2(U)}\le \Vert f\Vert _{L^1(U)} s. \end{aligned}$$

#### Proof

*Step 1.* We first argue that both *u* and $u^s$ are Hölder continuous functions for every $s>0$. This is implied by the De Giorgi-Nash-Moser theory since both $f \chi _{\{u>0\}}$ and $f \beta _s( u^s)$ belong to $L^q(U)$ with $q>\frac{d}{2}$. Notice also that $x \mapsto \beta _s( u^s(x))$ is Hölder continuous as well.

*Step 2.* We next show that $u^s$ is a nonnegative function, giving also (3.10). To this end, let $x_0~$ be a point of minimum for $u^s$ and suppose, on the contrary, that $u^s(x_0) < 0$. This implies that $x_0$ is an interior point of *U* since $g \ge 0$. By Hölder regularity of *u* and non-negativity of the boundary values, there exists positive $r=r(s, x_0)$ such that $u^s<0$ in $B_r(x_0)$. It thus follows that

$$\begin{aligned} \nabla \cdot (\textbf{b}\nabla u^s) =f \beta _s(u^s) <0 ~\text { a.e. in } ~ B_r(x_0), \end{aligned}$$

which means that $u^s$ is a continuous weak supersolution in $B_r(x_0)$ with an interior minimum point. By the strong minimum principle, implied by the weak Harnack inequality, we hence obtain that $u^s$ has to be a negative constant in $B_r(x_0)$, which violates the fact that $f \beta _s(u^s)<0$ a.e. through the equation; a contradiction. Therefore $u^s$ is nonnegative in *U* and, consequently, (3.10) is valid.

*Step 3.* We then show that (3.11) holds. Let $w^s:= u^s-u$. First we show that $w^s \le s$. For this, let $E:= \{ u^s> s \} \cap U$ and observe that if $u^s(x) \le s$ for some $x\in U$, then $w^s(x) \le s$ as well since $u \ge 0$. Thus $w_s \le s$ on $U {\setminus } E$. Moreover, $w^s \le s$ on $\partial E$ and

$$\begin{aligned} \nabla \cdot (\textbf{b}\nabla w^s)=f( 1- \chi _{\{u>0\}} )\ge 0~ \text { in }~ E. \end{aligned}$$

Therefore $w_s$ is a continuous subsolution in an open set *E* and it attains its maximum on the boundary of *E*, which implies $w^s \le s$ in the whole *E*. In conclusion, (3.11) is valid.

*Step 4.* We prove that $w^s = u^s-u \ge 0$. If $u(x)=0$ for some $x\in U$, then $w^s(x) \ge 0$ by Step 1. We thus need to show that $w^s \ge 0$ in the open set $V=\{ u> 0 \} \cap U$. Obviously, $w^s \ge 0$ on $\partial V$, and

$$\begin{aligned} \nabla \cdot (\textbf{b}\nabla w^s) =f( \beta _s(u^s) -1) \le 0 ~ \text { in } V, \end{aligned}$$

which means that $w^s$ is a weak supersolution in the set *V* and therefore it attains its minimum on the boundary of *V* and, therefore, $w^s \ge 0$.

*Step 5.* It remains to prove (3.12). This follows by testing the subtracted equations of $u^s$ and *u*, and from (3.11),

3.13 $$\begin{aligned} \Vert \nabla u^s-\nabla u\Vert ^2_{L^2(U)}&\le \int _U (\nabla u^s-\nabla u) \cdot \textbf{b}(\nabla u^s-\nabla u) \, \textrm{d}x \nonumber \\&\le \int _U f \left| \beta _s( u^s) -\chi _{\{u>0\}}\right| ( u^s- u) \, \textrm{d}x \nonumber \\&\le \Vert f\Vert _{L^1(U)} \Vert u-u^s\Vert _{L^{\infty }(U)} \le \Vert f\Vert _{L^1(U)} s. \end{aligned}$$

The proof is complete. $\square$

### Homogenization of the Penalized Obstacle Problem

We now prove the homogenization result for the penalized problem.

#### Proposition 3.4

Let *U* be a bounded Lipschitz domain. Let $q \in (d,\infty )$ and $\delta \in (0,\infty ]$. There exist constants $C(U,d,\Lambda )<\infty$ and $\alpha (\delta ,q,d,\Lambda ) \in (0,\tfrac{1}{2})$ such that the following statement is valid. Let $f \in L^q(U)$ be a given function such that $f > 0$ and $g \in W^{1,2+\delta }(U)$, $g \ge 0$. Let $\varepsilon \in (0,1]$ and $u^{s,\varepsilon }, u^s \in g+ W_0^{1,2}(U)$ solve

3.14 $$\begin{aligned} \nabla \cdot \left( \textbf{a}\left( \tfrac{\cdot }{\varepsilon }\right) \nabla u^{s,\varepsilon } \right) =f\beta _s(u^{s,\varepsilon }), \text { in } U, \end{aligned}$$

and3.15respectively. Then we have that3.16

#### Proof

Let $m = \lceil - \log _3 \varepsilon \rceil$. The idea of the proof is to compare $u^{s,\varepsilon }$ to the following function, so-called *two-scale expansion* of $u^{s}$, defined as

3.17 $$\begin{aligned} w^{s,\varepsilon } (x):=u^{s}(x)+\varepsilon \eta _r(x) \sum _{k=1}^{d}\partial _{x_k}(\zeta _h *u^{s} )(x) \phi _{m,e_k}\left( \tfrac{x}{\varepsilon }\right) . \end{aligned}$$

Here $\phi _{m,e}$ is the finite volume corrector corresponding to $\square _m$ and $e \in \mathbb {R}^d$ defined in (2.8). Moreover, $\zeta _h$ is a standard smooth mollifier supported in $B_h$ satisfying

$$\begin{aligned} \int _{B_h} \zeta _h = 1, ~ \Vert \nabla ^k \zeta _h\Vert \le C(k,d) h^{-k-d},~ k \in \mathbb {N}_0, \end{aligned}$$

and $\eta _r\in C_c^\infty (U)$ is a smooth cutoff function satisfying

$$\begin{aligned} 0\le \eta _r\le 1,~ \eta _r=1 \text { in } U_{2r},~ \eta _r \equiv 0 \text { in } U {\setminus } U_r, \quad | \nabla ^k \eta _r| \le C(k,d,U) r^{-k}, \end{aligned}$$

and $U_r:=\{x \in U; ~{{\,\textrm{dist}\,}}(x,\partial U)>r\}$. The parameters $h,r> 0$ will be chosen later on.

*Step 1.* Bounds for $u_s$ and for its convolution. First, since $f\beta _s({u^s}) \in L^q(U)$, we have by the standard Calderón–Zygmund theory that there exists $C(U,d,\Lambda ) <\infty$ such that, for every $r >0$,

3.18 $$\begin{aligned} \Vert \nabla ^2 u_s \Vert _{L^q(U_{r/2})} \le C r^{-d/2} \left( r^{-1} \Vert \nabla g \Vert _{L^2(U)} + \Vert f \Vert _{L^q(U)} \right) . \end{aligned}$$

Using Young’s inequality for convolutions, we have that, for every $h \in (0,r/2\wedge 1)$,

3.19 $$\begin{aligned} \left\| \nabla \big ( \eta _r \nabla (\zeta _h *u_s) \big ) \right\| _{L^\infty (U)}&\le C r^{-d/2} \left( r^{-1} \Vert \nabla g \Vert _{L^2(U)} + \Vert f \Vert _{L^q(U)} \right) \nonumber \\&\le C h^{-d/q} r^{-d/2} \left( r^{-1} \Vert \nabla g \Vert _{L^2(U)} + \Vert f \Vert _{L^q(U)} \right) . \end{aligned}$$

Moreover, using an elementary computation (see [7, Lemma 6.8]) we see that

3.20 $$\begin{aligned} \left\| \eta _r \nabla ( u^s - \zeta _h *u^s) \right\| _{L^2(U)}&\le C |U|^{1/2-1/q} h \Vert \nabla ^2 u \Vert _{L^q(U_{r/2})} \nonumber \\&\le C h^{1-d/q} r^{-d/2} \left( r^{-1} \Vert \nabla g \Vert _{L^2(U)} + \Vert f \Vert _{L^q(U)} \right) . \end{aligned}$$

Finally, by the Meyers’ estimate, see Lemma 2.6, we have higher global integrability for the gradient, namely that there exist constants $C(U,d,\Lambda )<\infty$ and $\sigma (d,\Lambda ) \in (0,\delta ]$ such that

$$\begin{aligned} \left\| \nabla u^s \right\| _{L^{2+\sigma }(U)} \le C \left( \Vert g \Vert _{W^{1,2+\delta }(U)} + \Vert f \Vert _{L^q(U)} \right) . \end{aligned}$$

which gives us, by Hölder’s inequality, that

3.21 $$\begin{aligned} \left\| \nabla u^s \right\| _{L^{2}(U {\setminus } U_{2r})} \le C r^{\frac{\sigma }{2+\sigma }} \left( \Vert g \Vert _{W^{1,2+\delta }(U)} + \Vert f \Vert _{L^q(U)} \right) . \end{aligned}$$

*Step 2.* We derive the following estimate

3.22 $$\begin{aligned} \Vert \nabla \cdot \left( \textbf{a}(\tfrac{\cdot }{\varepsilon }) \nabla w^{s,\varepsilon }\right) -f\beta _s(u^s) \Vert _{H^{-1}(U)} \le C \big ( \mathcal {E}(\varepsilon ) \big )^{\alpha } \left( \Vert g \Vert _{W^{1,2+\delta }(U)} + \Vert f \Vert _{L^q(U)} \right) \nonumber \\ \end{aligned}$$

with constants $C(U,d,\Lambda )<\infty$ and $\alpha (\delta ,d,\Lambda ) \in (0,1)$. By a direct computation, we get

$$\begin{aligned} \nabla w^{s,\varepsilon }&= \nabla (u^s - \zeta _h *u^s) + (1-\eta _r) \nabla \zeta _h *u^s \\&\quad + \eta _r \sum _{k=1}^d \big (e_k + \nabla \phi _{e_k}(\tfrac{\cdot }{\varepsilon }) \big ) \partial _{x_k} \zeta _h *u^s + \varepsilon \sum _{k=1}^d \phi _{e_k}(\tfrac{\cdot }{\varepsilon })\nabla \left( \eta _r \partial _{x_k}\zeta _h *u^s \right) . \end{aligned}$$

Since $\phi _{m,e_k}$ solves the equation $\nabla \cdot \textbf{a}(\tfrac{\cdot }{\varepsilon }) \big (e_k + \nabla \phi _{m,e_k}(\tfrac{\cdot }{\varepsilon }) \big ) = 0$, we obtain, by rearranging the terms, that

3.23 $$\begin{aligned} \nabla \cdot \left( \textbf{a}(\tfrac{\cdot }{\varepsilon }) \nabla w^{s,\varepsilon } \right)&= \sum _{k=1}^d \nabla ( \eta _r \partial _{x_k} \zeta _h *u^s) \cdot \textbf{a}(\tfrac{\cdot }{\varepsilon }) (e_k+ \nabla \phi _{m,e_k}(\tfrac{\cdot }{\varepsilon }) ) \nonumber \\&\qquad + \nabla \cdot \textbf{a}(\tfrac{\cdot }{\varepsilon }) \left( (1-\eta _r) \nabla u^s + \eta _r \nabla (u^s - \zeta _h *u^s) \right) \nonumber \\&\qquad + \varepsilon \sum _{k=1}^d \nabla \cdot \textbf{a}(\tfrac{\cdot }{\varepsilon }) \left( \phi _{m,e_k}(\tfrac{\cdot }{\varepsilon }) \nabla ( \eta _r \partial _{x_k} \zeta _h *u^s) \right) . \end{aligned}$$

On the other hand, using the fact that  has constant coefficients together with the equation for $u^s$, we deduce thatTherefore, we getFurthermore, we write  by means of the finite volume skew-symmetric flux corrector $\textbf{S}_{ij}^{e_k,m}$ defined in (2.9) so that, for $i \in \{1,\ldots ,d\}$,By skew-symmetry of $\textbf{S}_m^{(e_k)}$, we obtainIt follows thatEmploying the definition of $\mathcal {E}(\varepsilon )$ in , we finally derive

$$\begin{aligned}&\Vert \nabla \cdot \left( \textbf{a}(\tfrac{\cdot }{\varepsilon }) \nabla w^{s,\varepsilon } \right) -f\beta _s( u^s ) \Vert _{H^{-1}(U)} \\&\quad \le C \left( \mathcal {E}(\varepsilon ) \left\| \nabla (\eta _r \partial _{x_k}u^s) \right\| _{L^\infty (U)} + \left\| \nabla u^s \right\| _{L^{2}(U {\setminus } U_{2r})} + \left\| \eta _r \nabla ( u^s - \zeta _h *u^s) \right\| _{L^2(U)} \right) \\&\quad \le C \left( r^{-d/2-1} \big ( \mathcal {E}(\varepsilon ) h^{-d/q} + h^{1-d/q} \big ) + r^{\frac{\sigma }{2+\sigma }} \right) \left( \Vert g \Vert _{W^{1,2+\delta }(U)} + \Vert f \Vert _{L^q(U)} \right) . \end{aligned}$$

Optimizing in *h* and *r*, that is choosing $h = \mathcal {E}(\varepsilon ) \wedge \frac{1}{2}$ and $r = h^{\beta }$ with the exponent $\beta = \frac{q-d}{q} \big ( 1 + \frac{d}{2} + \frac{\sigma }{2+\sigma } \big )$, we obtain the desired inequality (3.22) with $\alpha = \frac{\beta \sigma }{2+\sigma }$.

*Step 3.* We show that

3.24 $$\begin{aligned} \Vert \nabla u^{s,\varepsilon } - \nabla w^{s,\varepsilon } \Vert ^2_{L^2(U)} \le C \Vert \nabla \cdot \textbf{a}^\varepsilon \nabla w^{s,\varepsilon }-f \beta _s( w^{s,\varepsilon } )\Vert _{H^{-1}(U)} \end{aligned}$$

By the ellipticity assumption and the fact that $u^{s,\varepsilon } - w^{s,\varepsilon } \in H_0^1(U)$, we get

$$\begin{aligned}&\Vert \nabla u^{s,\varepsilon } - \nabla w^{s,\varepsilon } \Vert ^2_{L^2(U)} \\&\quad \le \int _U \left( \nabla u^{s,\varepsilon } - \nabla w^{s,\varepsilon } \right) \cdot \textbf{a}\left( \tfrac{\cdot }{\varepsilon }\right) \left( \nabla u^{s,\varepsilon } - \nabla w^{s,\varepsilon } \right) \\&\quad \le - \int _U \left( u^{s,\varepsilon } - w^{s,\varepsilon } \right) f ( \beta _s( u^{s,\varepsilon }) - \beta _s( w^{s,\varepsilon } ) ) \\&\qquad + \Vert \nabla \cdot \left( \textbf{a}(\tfrac{\cdot }{\varepsilon }) \nabla w^{s,\varepsilon }\right) - f \beta _s( w^{s,\varepsilon } ) \Vert _{H^{-1}(U)} \Vert \nabla u^{s,\varepsilon } - \nabla w^{s,\varepsilon } \Vert _{L^{2}(U)} . \end{aligned}$$

Since the second term on the right is negative by the monotonicity of $\beta _s(\cdot )$, we obtain (3.24).

*Step 4.* It follows from (3.22) and (3.24), by the triangle inequality, that

$$\begin{aligned}&\Vert \nabla u^{s,\varepsilon } - \nabla w^{s,\varepsilon } \Vert ^2_{L^2(U)} \\&\quad \le C \Vert \nabla \cdot \left( \textbf{a}(\tfrac{\cdot }{\varepsilon }) \nabla w^{s,\varepsilon }\right) -f\beta _s(u^s) \Vert _{H^{-1}(U)} + C \Vert f \beta _s( w^{s,\varepsilon }) -f \beta _s( u^{s} )\Vert _{H^{-1}(U)} . \end{aligned}$$

We have, by Hölder’s inequality and the Sobolev inequality, that, for every $\psi \in H_0^1(U)$ with $\Vert \nabla \psi \Vert _{L^2(U)} \le 1$,

$$\begin{aligned} \int _U ( f \beta _s( w^{s,\varepsilon }) - f \beta _s( u^{s} )) \psi&\le \Vert f \Vert _{L^{d}(U)} \Vert \beta _s( w^{s,\varepsilon }) - \beta _s( u^{s} )\Vert _{L^{2}(U)} \Vert \psi \Vert _{L^{\frac{2d}{d-2}}(U)} \\&\le \frac{C}{s} \Vert f \Vert _{L^{d}(U)} \Vert w^{s,\varepsilon } - u^{s} \Vert _{L^{2}(U)} \Vert \nabla \psi \Vert _{L^{2}(U)} \\&\le \frac{C}{s} \Vert f \Vert _{L^{q}(U)} \Vert w^{s,\varepsilon } - u^{s} \Vert _{L^{2}(U)} . \end{aligned}$$

Furthermore, we have by the definitions of $w^{s,\varepsilon }~$and $\mathcal {E}(\varepsilon )$, together with (3.19), that

$$\begin{aligned} \Vert w^{s,\varepsilon } - u^{s} \Vert _{L^{2}(U)}&\le C \mathcal {E}(\varepsilon ) \Vert \eta _r \nabla (\zeta _h *u^{s}) \Vert _{L^{\infty }(U)} \\&\le C h^{-d/q} r^{-d/2} \mathcal {E}(\varepsilon ) \left( r^{-1} \Vert \nabla g \Vert _{L^2(U)} + \Vert f \Vert _{L^q(U)} \right) \\&\le C \bigl ( \mathcal {E}(\varepsilon ) \bigr )^{1-\alpha } \left( \Vert \nabla g \Vert _{L^2(U)} + \Vert f \Vert _{L^q(U)} \right) \,. \end{aligned}$$

It thus follows that

$$\begin{aligned} \Vert f \beta _s( w^{s,\varepsilon }) -f \beta _s( u^{s} )\Vert _{H^{-1}(U)} \le \frac{C}{s} \bigl ( \mathcal {E}(\varepsilon ) \bigr )^{1-\alpha } \Vert f \Vert _{L^q(U)} \left( \Vert \nabla g \Vert _{L^2(U)} + \Vert f \Vert _{L^q(U)} \right) \end{aligned}$$

The proof is complete by combining the above displays with (3.22) $\square$

We are ready to prove Theorem 1.1.

#### Proof of Theorem 1.1

We combine Lemma 3.3 with Proposition 3.4 by optimizing in *s*. Indeed, (3.12) yields that

$$\begin{aligned} \Vert \nabla u^{s,\varepsilon }-\nabla u^\varepsilon \Vert _{L^2(U)} + \Vert \nabla u^{s}-\nabla u \Vert _{L^2(U)} \le C \Vert f\Vert _{L^q(U)}^{\frac{1}{2}} s^{\frac{1}{2}} \end{aligned}$$

and (3.16) thatChoosing

$$\begin{aligned} \Vert f\Vert _{L^q(U)}^{1/2} s^{1/2}= &   s^{-1} \big ( \mathcal {E} (\varepsilon )\big )^{1-\alpha } \Vert f \Vert _{L^q(U)} \left( \Vert \nabla g \Vert _{L^{2+\delta }(U)} + \Vert f \Vert _{L^q(U)} \right) \\ \implies \quad s= &   \big ( \mathcal {E} (\varepsilon )\big )^{\frac{2}{3}(1-\alpha )} \Vert f \Vert _{L^q(U)}^{\frac{1}{3}} \left( \Vert \nabla g \Vert _{L^{2+\delta }(U)} + \Vert f \Vert _{L^q(U)} \right) ^{\frac{2}{3}} \end{aligned}$$

By redefining $\alpha$ to be the minimum $\alpha \wedge \frac{2}{3}(1-\alpha )$, we get that

$$\begin{aligned}  &   \Vert \nabla u^{s,\varepsilon }-\nabla u^\varepsilon \Vert _{L^2(U)} + \Vert \nabla u^{s}-\nabla u \Vert _{L^2(U)} \\  &   \quad \le C \big ( \mathcal {E} (\varepsilon )\big )^{\alpha } \left( \Vert \nabla g \Vert _{L^{2+\delta }(U)} + \Vert f \Vert _{L^q(U)} \right) \end{aligned}$$

andNow the result follows by the triangle inequality. $\square$

## Large-Scale $C^{1,1}$-Regularity of the Solution

In this section we prove Theorem 1.2, which says that the solution to normalized obstacle problem satisfies $C^{1,1}$-estimate on large-scales. Let $u^\varepsilon \ge 0$ solve the variational inequality

$$\begin{aligned} \nabla \cdot (\textbf{a}^\varepsilon \nabla u^\varepsilon ) =f \chi _{ \{ u^\varepsilon >0\} } \text { in } U. \end{aligned}$$

The idea of the proof is to combine boundedness of solutions close to the free boundary, provided by (2.31), together with the regularity of the equation $\nabla \cdot (\textbf{a}^\varepsilon \nabla u^\varepsilon ) =f$, proved in Lemma 4.2 below. Indeed, if $x_0\in \varGamma ({u^\varepsilon })$, then, by (2.31),

4.1 $$\begin{aligned} \sup _{B_r(x_0)} u^\varepsilon \le C \Vert f \Vert _{L^\infty } r^2, \end{aligned}$$

provided that $$ B_{2r} (x_0) \subseteq U$$. Here the constant $$C>0$$ depends on $$\Lambda , d$$. It is known that the quadratic decay bound above does not yet imply $$ C^{1,1}$$-regularity of solutions, in general, if *f* belongs merely to $$L^\infty (B_1)$$. In fact, even in the case of smooth coefficients, we need to impose further assumption on *f* in order to obtain $$C^{1,1}$$-regularity. Thus, we assume that *f* is Hölder continuous, which together with the homogenization then yields the large-scale $$C^{1,1}$$-regularity for $$ u^\varepsilon $$. The terminology “large-scale" stems from the rescaling of the problem. If we set $$R = \varepsilon ^{-1}$$, we may rescale $$u^\varepsilon $$ to be the solution in a very large ball $$B_{2R}$$:

$$\begin{aligned} \nabla \cdot (\textbf{a}\nabla u) = f(\varepsilon \cdot ) \chi _{\{u >0 \}} \quad \text{ in } B_{2R}. \end{aligned}$$

Next, recall definition of the linear space of -harmonic functions with controlled growth, (1.22),By the Liouville theorem, $\overline{\mathcal {A}}_k$ consists only of -harmonic polynomials of degree at most *k*. Recall also the definition in (1.23)

$$\begin{aligned} {\mathcal {A}}_k := \bigl \{ u \in H^1_{loc}( \mathbb {R}^d) \, : \, -\nabla \cdot (\textbf{a}\nabla u )=0, ~ \lim _{r\rightarrow \infty } r^{-k-1} \Vert u \Vert _{{\underline{L}^2(B_{r})}}=0 \bigr \}. \end{aligned}$$

As before, we use the notation $\psi ^\varepsilon = \psi (\tfrac{\cdot }{\varepsilon })$ for the rescaled members of $\mathcal {A}_k$.

Denote the minimal scale by

4.2 $$\begin{aligned} R_{\sigma ,\alpha } := \inf \bigl \{ r \in (0,\infty ) \, : \, \bigl ( \mathcal {E}\bigl ( \tfrac{\varepsilon }{r} \bigr )\bigr )^\alpha \le \sigma . \bigr \} \end{aligned}$$

The following proposition is essentially [7, Theorem 3.8]. Minor modifications are needed for its proof, but the changes are almost entirely typographical. If the reader wants to go through the proof very carefully, notice that (1.19) implies that, for every $\alpha \in (0,1)$,

4.3 $$\begin{aligned} \int _{r}^\infty \bigl ( \mathcal {E}\bigl ( \tfrac{\varepsilon }{s} \bigr )\bigr )^\alpha \, \frac{ds}{s} \le \frac{1}{\alpha \gamma } \bigl ( \mathcal {E}\bigl ( \tfrac{\varepsilon }{r} \bigr )\bigr )^\alpha \,. \end{aligned}$$

We omit the rest of the details of the proof.

### Proposition 4.1

Let $\gamma \in (0,1]$. Assume that $\mathcal {E}$ satisfies (1.19) and let $R_{\sigma ,\alpha }$ be defined in (4.2). There exist constants $\alpha ,\sigma \in (0,1]$ and $C< \infty$, all depending only on $(\gamma ,d,\Lambda )$, such that the following claims are valid.

- Let $k\in \mathbb {N}$ and $p \in \overline{\mathcal {A}}_k$. Then there exists $\psi \in \mathcal {A}_k$ such that, for every $r \in [ R_{\sigma ,\alpha },\infty )$,
  4.4 $$\begin{aligned} \left\| p - \psi ^\varepsilon \right\| _{\underline{L}^2 \left( B_{r} \right) } \le C \bigl ( \mathcal {E}\bigl ( \tfrac{\varepsilon }{r} \bigr )\bigr )^\alpha \left\| p \right\| _{\underline{L}^2 \left( B_{r} \right) } \end{aligned}$$
- Let $k\in \mathbb {N}$ and $\psi \in \mathcal {A}_k$. Then there exists $p \in \overline{\mathcal {A}}_k$ such that (4.4) holds for every $r \in [ R_{\sigma ,\alpha },\infty )$.
- If $u^\varepsilon$ solves $-\nabla \cdot \textbf{a}^\varepsilon \nabla u^\varepsilon = 0$ in $B_R$ with $R \ge R_{\sigma ,\alpha }$, then, for every $r \in [ R_{\sigma ,\alpha },R)$ and $k \in \mathbb {N}$, there exists $\psi \in \mathcal {A}_k$ such that
  4.5 $$\begin{aligned} \left\| u^\varepsilon - \psi ^\varepsilon \right\| _{\underline{L}^2 \left( B_{r} \right) } \le C\left( \frac{r}{R} \right) ^{k+1} \left\| u^\varepsilon \right\| _{\underline{L}^2 \left( B_{R} \right) } \end{aligned}$$

The next statement is the content of a much more general statement given in [7, Exercise 3.12] with $k = 1$. Since the proof is not entirely trivial, we present the proof for the sake of completeness.

### Lemma 4.2

Under the assumptions of Proposition 4.1, there exists a finite constant $C(\gamma ,d,\Lambda )$ such that if $R \in [ R_{\sigma ,\alpha },\infty )$ and $u^\varepsilon$ solves $\nabla \cdot \textbf{a}\nabla u^\varepsilon = f(\varepsilon \cdot ) \ge 0$, in $B_R$, then there exists $\psi \in \mathcal {A}_1$ such that, for every $r \in [ R_{\sigma ,\alpha },R]$,

$$\begin{aligned} \left\| u^\varepsilon - \psi ^\varepsilon \right\| _{\underline{L}^2 \left( B_{r} \right) } \le C\Bigl ( \frac{r}{R} \Bigr )^2 \Vert u^\varepsilon \Vert _{\underline{L}^2(B_{R})} + C R^2 \Vert f \Vert _{C^{0,\gamma }(B_R)} . \end{aligned}$$

### Proof

For given $r \in [ R_{\sigma ,\alpha },R]$ and $\psi _r \in \mathcal {A}_2$, let $w_r^\varepsilon , ~w_r$ solveLemma 3.1 implies that, for every $\tau \in (0,\tfrac{1}{2})$,

$$\begin{aligned} \Vert w_r^\varepsilon - w_r \Vert _{\underline{L}^2(B_{1/2})}&\le C \big (\mathcal {E}\bigl ( \tfrac{\varepsilon }{r} \bigr ) + \tau ^{\frac{\delta }{2+\delta } } \bigr ) \Vert \nabla w_r \Vert _{L^{1,2+\delta } (B_{1/2})} \\&\quad + C \mathcal {E}\bigl ( \tfrac{\varepsilon }{r} \bigr ) \big ( \tau ^{-1} \Vert \nabla w_r \Vert _{L^{\infty } (B_{\tau })} + \Vert \nabla ^2 w_r \Vert _{L^{\infty } (B_{\tau })} \big ) \,. \end{aligned}$$

Theorem 2.3 applied to $\nabla w_r$ and $\nabla ^2 w_r$, together with the Caccioppoli inequality, yields that

$$\begin{aligned} \tau ^{-1} \Vert \nabla w_r \Vert _{L^{\infty } (B_{\tau })} + \Vert \nabla ^2 w_r \Vert _{L^{\infty } (B_{\tau })} \le C \tau ^{-2 - \frac{d}{2} } \Vert w_r \Vert _{L^{2} (B_{1/2})} . \end{aligned}$$

Hence, by choosing $\tau$ appropriately by means of $\mathcal {E}\bigl ( \tfrac{\varepsilon }{r} \bigr )$ and applying Caccioppoli estimate for $u^\varepsilon - \psi ^\varepsilon$, we get that there exists a constant $\alpha (d,\Lambda ) \in (0,1)$ such that

$$\begin{aligned} r^2 \Vert w_r^\varepsilon - w_r \Vert _{\underline{L}^2(B_{1/2})} \le C \bigl ( \mathcal {E}\bigl ( \tfrac{\varepsilon }{r} \bigr ) \bigr )^\alpha \bigl ( \Vert u^\varepsilon - \psi _r^\varepsilon \Vert _{\underline{L}^{2} (B_{r})} + r^2 f(0) \bigr ) \,. \end{aligned}$$

Moreover, we may compare $w_r^\varepsilon$ and $u^\varepsilon - \psi _r^\varepsilon$ simply by testing as

$$\begin{aligned} \Vert u^\varepsilon - \psi ^\varepsilon _r - r^2 w_r^\varepsilon (r^{-1} \cdot ) \Vert _{\underline{L}^2(B_{r/2})} \le C r^{2+\gamma } [f]_{C^{0,\gamma }(B_1)}. \end{aligned}$$

Combining above inequalities thus yields that

4.6 $$\begin{aligned}&\Vert u^\varepsilon - \psi ^\varepsilon _r - r^2 w_r(r^{-1} \cdot ) \Vert _{\underline{L}^2(B_{r/2})} \nonumber \\&\quad \le C \bigl ( \mathcal {E}\bigl ( \tfrac{\varepsilon }{r} \bigr ) \bigr )^\alpha \bigl ( \Vert u^\varepsilon - \psi _r^\varepsilon \Vert _{\underline{L}^{2} (B_{r})} + r^2 f(0) \bigr ) + C r^{2+\gamma } [f]_{C^{0,\gamma }(B_1)} \,. \end{aligned}$$

Next, since $w_r - q$ is an -harmonic function, we deduce that, for every $s \in (0,\tfrac{1}{2} r)$,

4.7 $$\begin{aligned} \inf _{ p \in \overline{\mathcal {A}}_2}\Vert r^2 w_r(r^{-1} \cdot ) - q - p \Vert _{\underline{L}^2(B_{s})} \le C \Bigl ( \frac{s}{r} \Bigr )^3 \Vert r^2 w_r(r^{-1} \cdot ) - q \Vert _{\underline{L}^2(B_{r})} \,. \end{aligned}$$

Letting $\widetilde{p}_s \in \overline{\mathcal {A}}_2$ be the minimizing polynomial for the infimum on the left, we see that $\widetilde{p}_s$ satisfies, by the triangle inequality, that

4.8 $$\begin{aligned} \left\| {\widetilde{p}}_s \right\| _{\underline{L}^2 \left( B_{s} \right) }&\le \Vert r^2 w_r(r^{-1} \cdot ) - q \Vert _{\underline{L}^2(B_{s})} \le C \Bigl ( \frac{s}{r} \Bigr )^{-d/2} \Bigl ( \Vert u^\varepsilon - \psi _r^\varepsilon \Vert _{\underline{L}^{2} (B_{r})} + r^2 f(0) \Bigr ) . \end{aligned}$$

Thus, by choosing $s = \theta r$ with $\theta (d,\Lambda ) \in (0,1)$ small enough, the triangle inequality yields that

$$\begin{aligned}&\Vert u^\varepsilon {-} \psi _r^\varepsilon {-} q {-} \widetilde{p}_{\theta r} \Vert _{\underline{L}^2(B_{\theta r})} \le \frac{1}{2} \theta ^2 \Vert u^\varepsilon {-} \psi _r^\varepsilon {-} q \Vert _{\underline{L}^2(B_{r})} \\&\quad + C \bigl ( \mathcal {E}\bigl ( \tfrac{\varepsilon }{r} \bigr ) \bigr )^\alpha \bigl ( \Vert u^\varepsilon - \psi _r^\varepsilon \Vert _{\underline{L}^{2} (B_{r})} + r^2 f(0) \bigr ) + C r^{2+\gamma } [f]_{C^{0,\gamma }(B_1)} \, . \end{aligned}$$

Furthermore, by Proposition 4.1 and (4.8), we find $\widetilde{\psi }_{\theta r} \in \mathcal {A}_2$ such that

$$\begin{aligned} \Vert {\widetilde{\psi }}_r^\varepsilon - \widetilde{p}_{\theta r} \Vert _{\underline{L}^2(B_{\theta r})} \le C \bigl ( \mathcal {E}\bigl ( \tfrac{\varepsilon }{r} \bigr )\bigr )^\alpha \bigl ( \Vert u^\varepsilon - \psi _r^\varepsilon \Vert _{\underline{L}^{2} (B_{r})} + r^2 f(0) \bigr ) . \end{aligned}$$

Denoting hence $\psi _{\theta r} = \psi _r + \widetilde{\psi }_{\theta r}$ and taking $\sigma$ so small that $C \bigl ( \mathcal {E}\bigl ( \tfrac{\varepsilon }{r} \bigr ) \bigr )^\alpha \le \frac{1}{4} \theta ^2$, we arrive at the inequality

$$\begin{aligned} \frac{\Vert u^\varepsilon {-} \psi _{\theta r}^\varepsilon {-} q \Vert _{ \underline{L}^2(B_{\theta r})}}{\theta ^2 r^2} \le \frac{1}{2} \frac{ \Vert u^\varepsilon {-} \psi _r^\varepsilon {-} q \Vert _{\underline{L}^2(B_{r})}}{r^2} + C \bigl ( \mathcal {E}\bigl ( \tfrac{\varepsilon }{r} \bigr ) \bigr )^\alpha f(0) + C r^{\gamma } [f]_{C^{0,\gamma }(B_1)} \,. \end{aligned}$$

Integrating this against the Haar measure by using the previous inequality and using (4.3), we arrive after reabsorption at

$$\begin{aligned} \int _{ R_{\sigma ,\alpha }}^R \inf _{\psi \in \mathcal {A}_2}\Vert u^\varepsilon {-} \psi ^\varepsilon {-} q \Vert _{\underline{L}^2(B_{t})} \, \frac{\textrm{d}t}{t^3} \le C \Vert u^\varepsilon \Vert _{\underline{L}^2(B_{1})} + C \Vert f \Vert _{C^{0,\gamma }(B_1)} \,. \end{aligned}$$

Letting then $\psi _r \in \mathcal {A}_2$ realize the infimum above for the radius *r*, we obtain by the triangle inequality that

$$\begin{aligned} r^{-2}\Vert \psi _r^\varepsilon - \psi _{2r}^\varepsilon \Vert _{\underline{L}^2(B_{r})} \le C \int _{r/2}^{3r} \inf _{\psi \in \mathcal {A}_2}\Vert u^\varepsilon {-} \psi ^\varepsilon {-} q \Vert _{\underline{L}^2(B_{t})} \, \frac{\textrm{d}t}{t^3} \end{aligned}$$

Therefore, by telescoping and using the quadratic growth in the form $\Vert \psi _r^\varepsilon - \psi _{2r}^\varepsilon \Vert _{\underline{L}^2(B_{s})} \le C (\tfrac{s}{r})^{2} \Vert \psi _r^\varepsilon - \psi _{2r}^\varepsilon \Vert _{\underline{L}^2(B_{r})}$ for every $s \in [r,1]$, and by denoting ${\widetilde{\psi }} = \psi _{R_{\sigma ,\alpha }}$, we deduce that

$$\begin{aligned} \Vert u^\varepsilon - {\widetilde{\psi }}^\varepsilon \Vert _{\underline{L}^2(B_{r})} \le C\Bigl ( \frac{r}{R} \Bigr )^2 \Vert u^\varepsilon \Vert _{\underline{L}^2(B_{R})} + C R^2 \Vert f \Vert _{C^{0,\gamma }(B_R)} . \end{aligned}$$

Finally, we remove $\mathcal {A}_2\backslash \mathcal {A}_1$ part from ${\widetilde{\psi }}$. For this, we first find, by Proposition 4.1, a polynomial $p_\psi = p_2 + p_1$, with $p_2 = \frac{1}{2} x \cdot \nabla ^2 p_\psi x$ and $p_1 = p_\psi - p_2$, tracking down $\psi$ so that

$$\begin{aligned} \Vert {\widetilde{\psi }}^\varepsilon - p_\psi \Vert _{\underline{L}^2(B_{r})} \le C \bigl ( \mathcal {E}\bigl ( \tfrac{\varepsilon }{r} \bigr )\bigr )^\alpha \Vert p_\psi \Vert _{\underline{L}^2(B_{r})} \end{aligned}$$

Letting $\psi _2$ be given by Proposition 4.1 tracking down $p_2$, we see that it has to have quadratic growth. In particular,

$$\begin{aligned}&\Vert \psi _2^\varepsilon \Vert _{\underline{L}^2(B_{r})} \le 2\Vert p_2 \Vert _{\underline{L}^2(B_{r})} = 2\Bigl ( \frac{r}{R} \Bigr )^2 \Vert p_2 \Vert _{\underline{L}^2(B_{R})} \le 2 \Bigl ( \frac{r}{R} \Bigr )^2 \Vert p_\psi \Vert _{\underline{L}^2(B_{R})} \\&\quad \le 4 \Bigl ( \frac{r}{R} \Bigr )^2 \Vert \psi ^\varepsilon \Vert _{\underline{L}^2(B_{R})} \,. \end{aligned}$$

Thus we deduce that

$$\begin{aligned} \Vert \psi _2^\varepsilon \Vert _{\underline{L}^2(B_{r})} \le C\Bigl ( \frac{r}{R} \Bigr )^2 \Vert u^\varepsilon \Vert _{\underline{L}^2(B_{R})} + C R^2 \Vert f \Vert _{C^{0,\gamma }(B_R)} . \end{aligned}$$

Finally, it is easy to see that $\psi = {\widetilde{\psi }} - \psi _2$ belongs to $\mathcal {A}_1$ and this is the corrector of the statement. The proof is complete. $\square$

### Proof of Theorem 1.2

There are three cases. The first case is that there is $R \in [R_{\sigma ,\alpha },\tfrac{1}{2}]$ such that there is a free boundary point $x_0$ in $B_{2R}$, but $\varGamma (u^\varepsilon ) \cap B_R = \emptyset$. Then (2.31) implies that, for every $s \in [R,1]$,

$$\begin{aligned} \Vert u^\varepsilon \Vert _{L^\infty (B_{s})} \le C s^2 \Vert f \Vert _{L^\infty (B_1)}. \end{aligned}$$

This, in particular, gives the bound in $L^\infty (B_{R})$. Applying next Lemma 4.2 in $B_R$ yields that there exists $\psi \in \mathcal {A}_1$ such that, for every $r \in [R_{\sigma ,\alpha },R]$,

$$\begin{aligned} \Vert u^\varepsilon - \psi ^\varepsilon \Vert _{\underline{L}^2(B_{r})} \le C\Bigl ( \frac{r}{R} \Bigr )^2 \Vert u^\varepsilon \Vert _{\underline{L}^2(B_{R})} + C R^2 \Vert f \Vert _{C^{0,\gamma }(B_R)} . \end{aligned}$$

Combining the previous two displays gives us, for $r \in [R_{\sigma ,\alpha },R]$,

$$\begin{aligned} \Vert u^\varepsilon - \psi ^\varepsilon \Vert _{\underline{L}^2(B_{r})} \le C r^2 \Vert f \Vert _{L^\infty (B_1)} \end{aligned}$$

Now, since $\psi$ has linear growth, we obtain that, for every $s \in [R,1]$,

$$\begin{aligned}&\Vert \psi ^\varepsilon \Vert _{\underline{L}^2(B_{s})} \le C \frac{s}{R} \Vert \psi ^\varepsilon \Vert _{\underline{L}^2(B_{R/2})} \le C \frac{s}{R} \Bigl ( \Vert u^\varepsilon \Vert _{\underline{L}^\infty (B_{R})} {+} \Vert u^\varepsilon - \psi ^\varepsilon \Vert _{\underline{L}^2(B_{R/2})} \Bigr ) \\&\quad \le Cs^2 \Vert f \Vert _{L^\infty (B_1)}, \end{aligned}$$

and thus (1.24) holds in this case.

The second case is that there is a free boundary point of *u* in $B_{R_{\sigma ,\alpha }}$. In this case (1.24) follows directly from (2.31).

Finally, the third case is that $\varGamma (u )\cap B_1 = \emptyset$. In the case we may directly apply Lemma 4.2 for $R = 1$ and deduce that (1.24) is valid with $\underline{L}^2$-norm on the left instead of $L^\infty$. However, we can apply Theorem 2.3 to upgrade the integrability. The proof is complete. $\square$

## Homogenization and Large-Scale Regularity of the Free Boundary

In this section we discuss the regularity of the free boundary of the normalized heterogeneous problem. Throughout this section $\lambda \in (0,\infty )$ is fixed. Let $u^\varepsilon ~$ and *u* be solutions to the normalized obstacle problem, respectively, with coefficients $\textbf{a}^\varepsilon$ and , with $f \equiv \lambda$, and recall that $\varGamma ( u^\varepsilon )$ and $\varGamma (u)$ denote the corresponding free boundaries.

Our goal is to answer the following two questions;

1. How close is $\varGamma (u^\varepsilon )$ to $\varGamma (u)$?
2. What can we say about the regularity of $\varGamma ( u^\varepsilon )$?

The discussion of the regularity of the free boundary for the homogenized obstacle problem evolves by studying the rescaled solutions around free boundary points, for $x_0 \in \varGamma (u)$, denote $u_{r, x_0} = \tfrac{u(rx +x_0)}{r^2}$. Geometrically, we zoom around a free boundary point, and investigate the limit as $r \rightarrow 0+$. By classifying the blow-ups and showing their uniqueness at a given free boundary point, we understand the local behavior of the free boundary. Furthermore, if we quantify the rate of the convergence to the blow-up solution as $r \rightarrow 0+$, we automatically obtain local estimates on the regularity of the free boundary.

The argument described above is no longer applicable, when we are given merely bounded measurable coefficients. Indeed, let $\varepsilon >0$ be a fixed number, and $x_0 \in \varGamma (u^\varepsilon )$ be a free boundary point. Denote by

5.1 $$\begin{aligned} u^\varepsilon _{r,x_0}(x):=\frac{u^\varepsilon (rx+x_0)}{r^2}, \text { where} ~r< {{\,\textrm{dist}\,}}(x_0, \partial B_1). \end{aligned}$$

Then $u^\varepsilon _{r,x_0} \ge 0$ solves the following obstacle problem

5.2 $$\begin{aligned} \nabla \cdot (\textbf{a}^\varepsilon _{x_0,r} \nabla u^\varepsilon _{r,x_0}(x)) =\lambda \chi _{\{u^\varepsilon _{r,x_0}>0 \}}, \end{aligned}$$

where

$$\begin{aligned} \textbf{a}_{x_0,r} :=\textbf{a}\left( {rx+x_0}\right) ~ \text { and }~ \textbf{a}^\varepsilon _{x_0,r} :=\textbf{a}\left( \tfrac{rx+x_0}{\varepsilon }\right) . \end{aligned}$$

The matrix $\textbf{a}(rx+x_0)$ is uniformly elliptic with the given constants.

For a moment, assume that $\textbf{a}$ has continuous entries, i.e.  $\textbf{a}^\varepsilon _{x_0,r} \rightarrow \textbf{a}^\varepsilon ({x_0})$ as $r\rightarrow 0$. If $u^\varepsilon _{r_j,x_0} \rightarrow u_0^\varepsilon$ as $r_j\rightarrow 0+$, then $u^\varepsilon _0 \ge 0$ is a global solution to the following obstacle problem

5.3 $$\begin{aligned} \nabla \cdot (\textbf{a}^\varepsilon ({x_0}) \nabla u^\varepsilon _{0}) =\lambda \chi _{\{u^\varepsilon _{0}>0\}}. \end{aligned}$$

If $u^\varepsilon _0$ is a half-space solution of the form

5.4 $$\begin{aligned} u_0^\varepsilon (x)=\lambda \frac{(x\cdot e)_+^2}{2e\cdot \textbf{a}^\varepsilon (x_0)e}, \end{aligned}$$

where $e \in {\mathbb {R}^d}$ is a unit vector, then $x_0$ is called a regular free boundary point in a usual sense. If $u^\varepsilon _{r,x_0} \rightarrow u_0^\varepsilon$ as $r\rightarrow 0+$, then *e* is the approximate unit normal vector to $\varGamma (u^\varepsilon )$ at $x_0$.

In a more general situation, without additional assumptions imposed on the matrix $\textbf{a}$, we cannot define the blow-up solution in the form (5.4). Instead we rely on an alternative definition for regular free boundary points, based on the density of the coincidence set $\varLambda (u^\varepsilon )$, see Definition 1.4.

A closely related quantity in the analysis of the free boundaries is so-called minimum diameter of a set.

### Definition 5.1

Given $U \subseteq \mathbb {R}^d$, *the minimum diameter* of *U*, denoted ${{\,\textrm{md}\,}}(U)$, is the infimum among the distances between pairs of parallel hyperplanes enclosing *U*.

Notice that we have the following trivial lower bound for the minimum diameter by means of the density:

5.5 $$\begin{aligned} 2 \ge \frac{{{\,\textrm{md}\,}}(\varLambda \cap B_r) }{r } \ge \frac{ |\varLambda \cap B_r|}{ d| B_r|}. \end{aligned}$$

Caffarelli’s alternative in measure form, [16, 17], says that if $\textbf{a}\in VMO$, then there exists

5.6 $$\begin{aligned} \lim _{r \rightarrow 0+} \frac{| \varLambda (u) \cap B_r(x_0)|}{|B_r(x_0)|} =\frac{1}{2}, \end{aligned}$$

and $$ x_0$$ is a regular point, or otherwise

5.7 $$\begin{aligned} \lim _{r \rightarrow 0+} \frac{ | \varLambda (u) \cap B_r(x_0)|}{|B_r(x_0)|} =0, \end{aligned}$$

and $$ x_0$$ is a singular point. Let us mention that when , a stronger result holds: for any $$\theta >0$$ small there exists an $$ 0<r_0<1$$ and $$0<\tau <1$$ such that if for some $$ t < r_0$$

$$\begin{aligned} \frac{| \varLambda (u) \cap B_t(x_0)|}{|B_t(x_0)|} \ge \theta ,~ \text { then}~~ \inf _{0<r\le \tau t} \frac{| \varLambda (u) \cap B_r(x_0)|}{|B_r(x_0)|} \ge \frac{1}{2}-\theta , \end{aligned}$$

where the parameters $r_0, \tau$ are universal, i.e. they depend on the given parameters, but not on the solution *u*.

In the next easy example, we see that the Caffarelli’s alternative does not hold, when $\textbf{a}$ is discontinuous, since we may have a contact set, with density $\frac{1}{4}$ at a free boundary point.

### Example

Let in dimension 2, $$ \textbf{a}= I $$ in $$ (0,1) \times (0,1)\cup (-1,0) \times (-1,0)$$, and $$ \textbf{a}= 2I $$ in $$ (-1,0) \times (0,1) \cup (-1,0)\times (0,1) $$. Then $$ u^0= x^2\chi _{\{ x>0\}}+y^2 \chi _{ \{ y>0\}} $$, solves the obstacle problem $$ \nabla \cdot (\textbf{a}\nabla u) =4 \chi _{ \{u>0\} }$$, in $$(-1,1)\times (-1,1)$$, and the contact set $$\{ u^0 \equiv 0\}$$ has density $$ \frac{1}{4}$$ at the origin, the free boundary has a corner point. In other words, the blow-up of $$ u^0$$ at the origin is a homogeneous global solution, which is neither one-dimensional, nor a polynomial.

### Qualitative Flatness of the Free Boundary

In this manuscript we consider the set of half-space solutions for the homogenized obstacle problem,5.8and the space of homogeneous half-space solutions, $$ \mathbb {H}^0$$, defined in (1.25), when $$ t=0$$.

The next result states that the free boundary is flat around a regular free boundary point. The result is qualitative in nature since we may choose the parameter $\delta$ measuring the flatness very small, but then we do not have quantitative control of the constants appearing in the proof. Quantification will be given in the next section.

#### Proposition 5.2

Let $\delta ,\vartheta \in (0,1/2)$. There exist constants $r_0,\gamma \in (0,1)$ and $\sigma \in (0,\delta ]$, all depending only on $(\delta ,\vartheta ,\lambda ,d,\Lambda )$, and $\alpha (d,\Lambda ) \in (0,1)$ such that the following claim is valid. Let $u^\varepsilon$ solve the equation

$$\begin{aligned} \nabla \cdot \textbf{a}^\varepsilon \nabla u^\varepsilon = \lambda \chi _{\{ u^\varepsilon > 0 \} } \qquad \text{ in } B_1\,. \end{aligned}$$

Suppose that $0 \in \varGamma (u^\varepsilon )$ and that the following conditions are valid: For some $r \in (0,r_0]$ we have that

5.9 $$\begin{aligned} \inf _{ s \in (\gamma r, \gamma ^{-1} r)} \frac{|\Lambda (u^\varepsilon ) \cap B_s |}{|B_s|} \ge \vartheta \quad \text{ and } \quad \big ( \mathcal {E}(\varepsilon )\big )^\alpha \le \sigma r^2 \end{aligned}$$

Then

5.10 $$\begin{aligned} \inf _{h \in \mathbb {H}^0} \Vert u^\varepsilon -h \Vert _{L^\infty (B_{r})} \le \delta r^2 \,. \end{aligned}$$

To prove the above proposition, we consider solutions $u^\varepsilon$ and *u* of the equations5.11We obtain the estimate for $\Vert u^\varepsilon -u \Vert _{L^\infty }$ using Theorem 1.1, Lemma 2.6 and the interpolation Lemma 2.1. Indeed, there exist constants $C(\lambda ,d,\Lambda )<\infty$ and $\alpha (d,\Lambda )\in (0,1)$ such that

5.12 $$\begin{aligned} \Vert u^\varepsilon -u \Vert _{L^\infty (B_{1/2})} \le C \bigl ( \mathcal {E}(\varepsilon ) \bigr )^\alpha ( \Vert u \Vert _{L^2(B_1)} + \Vert u^\varepsilon \Vert _{L^2(B_1)} + \lambda ) . \end{aligned}$$

The first lemma we prove states that if the origin is a free boundary point of $u^\varepsilon$ and the homogenized solution is close-by pointwise, then there exists a free boundary point of the homogenized problem close to origin as well.

#### Lemma 5.3

(Closeness of free boundaries) Let $\eta \in (0,1)$. There exists a constant $\theta (\eta ,\lambda ,d,\Lambda ) \in (0,1)$ such that the following claim is valid. Let $u^\varepsilon$ and *u* solve (5.11). Suppose further that $0 \in \varGamma (u^\varepsilon )$ and that, for some $s\in (0,1/2)$, we have that both

5.13 $$\begin{aligned} {{\,\textrm{md}\,}}(\varLambda (u^\varepsilon ) \cap B_{\theta s}) \ge \eta \theta s \end{aligned}$$

and

5.14 $$\begin{aligned} \Vert u^{\varepsilon } - u \Vert _{L^\infty (B_{1/2})} \le \theta ^4 s^2 \end{aligned}$$

are valid. Then there is a free boundary point of *u* in $B_s$, that is, $\varGamma (u) \cap B_s \ne \emptyset$.

#### Proof

Suppose that $0 \in \varGamma (u^\varepsilon )$ and assume that (5.13) and (5.14) are valid with $s\in (0,1/2)$ and with $\theta$ to be fixed. Without loss of generality, we may assume that $u^\varepsilon \ne u$ in $B_1$. We claim that $\varGamma (u) \cap B_s$ is non-empty. Assume, on the contrary, that $\varGamma (u) \cap B_s = \emptyset$. There are then two cases.

*Case 1:* $B_s \subseteq \varLambda (u)$. This is the easy case by the non-degeneracy. Indeed, we have

$$\begin{aligned} c s^2 \le \sup _{B_s} u^{\varepsilon } = \sup _{B_s} (u^{\varepsilon } - u) \le \Vert u^{\varepsilon } - u \Vert _{L^\infty (B_{1/2})} \le \theta ^4 s^2 \,, \end{aligned}$$

which gives a contradiction provided that $\theta$ is small enough.

*Case 2:* $B_s \subseteq \{ u>0 \}$. In the second case we need to use more technical argument appealing to the regularity of *u*. Since  in $B_s$, using the bound on $u^\varepsilon$ giving

$$\begin{aligned} \Vert u \Vert _{L^\infty (B_s)} \le \Vert u^\varepsilon \Vert _{L^\infty (B_s)} + \Vert u^\varepsilon - u \Vert _{L^\infty (B_{1/2})} \le Cs^2 + \theta ^2 s^2 \le Cs^2, \end{aligned}$$

we deduce by the -harmonicity of  that

$$\begin{aligned} \Vert \nabla ^2 u \Vert _{L^\infty (B_{s/2}) } + s \Vert \nabla ^3 u \Vert _{L^\infty (B_{s/2}) } \le C \,. \end{aligned}$$

Therefore, we obtain, with the affine function $\ell _y(x) = u(y) + \nabla u(y) \cdot {(x-y)}$ and quadratic polynomial $p_y(x) = \frac{1}{2} (x-y) \cdot \nabla ^2 u(y) (x-y)$, that, for every $t \in (0,s/4]$ and $y \in B_{s/4}$,

$$\begin{aligned} \Vert u - \ell _y - p_y \Vert _{L^\infty (B_t(y))} \le Ct^2 \frac{t}{s} \,. \end{aligned}$$

Notice that . Suppose then that $y \in \varLambda (u^\varepsilon )$ and take $t =\Vert u^\varepsilon - u \Vert _{L^\infty (B_{1/2})}^{1/2}$. Then $|u(y)| \le t^2$. Moreover, since $u^\varepsilon (y) = 0$, we have by (2.7) that

$$\begin{aligned} \Vert u \Vert _{L^\infty (B_t(y))} \le \Vert u^\varepsilon - u \Vert _{L^\infty (B_{1/2})} + \Vert u^\varepsilon \Vert _{L^\infty (B_t(y))} \le t^2 + C t^2 \le Ct^2 \,. \end{aligned}$$

If $\nabla u(y) \ne 0$, we apply the above estimates at $x^* = y + t \nabla u(y)/|\nabla u(y)|$ to obtain

$$\begin{aligned}&|\nabla u(y)| =t^{-1} | \ell _y(x^*)- u(y)|\\&\quad \le t^{-1}\bigl ( | \ell _y(x^*) - u(x^*)| + | u(x^*)- u(y) | \bigr ) \le Ct \le C \theta (\theta s) \,. \end{aligned}$$

Furthermore, since $u - \ell _0 - p_0$ is -harmonic, we deduce that

$$\begin{aligned} \Vert \nabla u - \nabla u(0) -\nabla p_0 \Vert _{L^\infty (B_{\theta s})} \le \frac{C}{\theta s} \Vert u - \ell _0 - p_0 \Vert _{L^\infty (B_{2\theta s})} \le C \theta (\theta s)\,. \end{aligned}$$

The above two displays yield the bound

5.15 $$\begin{aligned} \Vert \nabla p_0 \Vert _{L^\infty (\varLambda (u^\varepsilon ) \cap B_{\theta s})} \le C \theta (\theta s)\,. \end{aligned}$$

Next, the assumption (5.13) on the minimal distance implies that there are at least *d* points in $\varLambda (u^\varepsilon ) \cap B_{\theta s}$, say $\{y_j\}_{j=1}^{d} \subseteq \varLambda (u^\varepsilon ) \cap B_{\theta s}$, such that the matrix *Z* with columns $z_j = (\theta s)^{-1} y_j$, $j \in \{1,\ldots ,d\}$, has the modulus of the determinant bounded below by $2^{-d} \eta ^d$. Indeed, the origin belongs to $U := (\theta s)^{-1} \varLambda (u^\varepsilon ) \cap B_{1}$ and ${{\,\textrm{md}\,}}(U)\ge \vartheta$ by (5.13). Therefore, there must be a point $z_1$ with $|z_1| \ge \frac{1}{2} \vartheta$. Let then $n \in \mathbb {N}$, $n < d$, and suppose that we have, for every $k \in \{1,\ldots ,n\}$, $z_k \in U$ and a hyperplane $H_k = {{\,\textrm{span}\,}}\{z_1,\ldots ,z_k\}$ and projections $P_k$ onto $H_k$ and $P_k^\perp = I_d-P_k$ onto the orthogonal complement of $H_k$ such that $\alpha _{k} := |P_{k-1}^\perp z_k| \ge \frac{1}{2} \vartheta$. For $n=1$ this is valid trivially, because we may take $P_0 = 0$ and $P_1 = |z_1|^{-2} z_1 \otimes z_1$. Now, since ${{\,\textrm{md}\,}}(U)\ge \vartheta$, we thus find $z_{n+1} \in U$ such that $\alpha _{n+1} := |P_{n}^\perp z_{n+1}| \ge \frac{1}{2} \vartheta$. Inductively we hence find $\{z_1,\ldots ,z_d \}$ with aforementioned properties.

We then check that $\det (Z) \ge 2^{-d} \vartheta ^d$. For this, set $Z_k = [z_1 , \ldots , z_k ]$ and compute

$$\begin{aligned} \det (Z)&= \big | [Z_{d-1} , z_d] \big | = \big | [Z_{d-1}, P_{d-1}^\perp z_d] \big | + \big | [Z_{d-1}, P_{d-1} z_d]. \end{aligned}$$

The last determinant is zero since $P_{d-1} z_d \in H_{d-1}$. Furthermore,

$$\begin{aligned} \big | [Z_{d-1}, P_{d-1}^\perp z_d] \big | = \big | [Z_{d-2}, P_{d-2}^\perp z_{d-1} , P_{d-1}^\perp z_d] \big | + \big | [Z_{d-2}, P_{d-2} z_{d-1} , P_{d-1}^\perp z_d] \big |\,, \end{aligned}$$

and again the last determinant is zero. Continuing using the same principle we deduce that

$$\begin{aligned} \det (Z) = \big | [z_1, P_{1}^\perp z_{2} , \ldots , P_{d-1}^\perp z_d] \big |\,. \end{aligned}$$

The projections have the property that $P_k P_m = P_k$ for every $k > m$, which implies, by the fact that $z_{k+1} = P_{m} z_{k+1}$, that

$$\begin{aligned}&P_k^\perp z_{k+1} \cdot P_m^\perp z_{m+1} = z_{k+1} \cdot (I_d - P_k - P_m + P_k P_m) z_{m+1} \\&\quad = P_{m} z_{k+1} \cdot (I_d - P_m) z_{m+1} = 0 \,. \end{aligned}$$

It thus follows that

$$\begin{aligned} \det (Z) = |z_1| \prod _{k=1}^{d-1} |P_k^\perp z_{k+1}| \ge 2^{-d} \vartheta ^d\,, \end{aligned}$$

as claimed. The determinant bound yields that $| Z^{-1}| \le (\det Z)^{-1} \textrm{Adj}(Z) \le C(d) \vartheta ^{-d}$.

Applying now (5.15), we deduce that

$$\begin{aligned} | \nabla ^2 u(0) z_k | = \frac{1}{\theta s} | \nabla p_0(y_k) | \le \frac{1}{\theta s} \Vert \nabla p_0 \Vert _{L^\infty (\varLambda (u^\varepsilon ) \cap B_{\theta s})} \le C \theta \,. \end{aligned}$$

It follows that

$$\begin{aligned} | \nabla ^2 u(0)| = | \nabla ^2 u(0) Z Z^{-1}| \le | \nabla ^2 u(0) Z| |Z^{-1}| \le C \theta \vartheta ^{-d} \,, \end{aligned}$$

which then contradicts the fact that  for $\theta \le (2Cd \Lambda )^{-1} \lambda \vartheta ^{d}$.

The proof is now complete since we have shown that neither Case 1 nor Case 2 is possible, implying that there must be a free boundary point of *u* in $B_s$. $\square$

With the aid of the previous lemma we are able to find free boundary points of *u* close to the origin. After a linear change of variables, we may then consider the problem $\Delta v = \lambda \chi _{\{ v>0 \}}$ and show the closeness to a half-plane solution. The following lemma is essentially a consequence of [19, Lemma 6].

#### Lemma 5.4

([19]) Let $\rho , \eta \in (0,10^{-2})$. There exist constants $\tau ,s_0 \in (0,1)$ depending only on $(\rho , \eta ,d)$ such that if  *u* is a solution of $\Delta u =\lambda \chi _{ \{ u>0\}}$ in $B_1$ such that $0 \in \varGamma (u)$, ${{\,\textrm{md}\,}}(\varLambda (u) \cap B_s) \ge \eta s$ for some $s \in (0,s_0]$, then there exists $e \in \partial B_1$ such that

$$\begin{aligned} \{ x \in B_{\tau s} \, : \, e\cdot x \le - \rho \tau s \} \subseteq \varLambda (u) \quad \text{ and } \quad \{ x \in B_{\tau s} \, : \, e\cdot x \ge \rho \tau s \} \subseteq \{ u > 0 \} \,. \end{aligned}$$

Moreover, there exists a constant $C(\lambda ,d) < \infty$ such that

5.16 $$\begin{aligned} \inf _{e \in \partial B_1} \sup _{x \in B_{\tau s/12}} \Big | u(x) - \frac{\lambda }{2} (e\cdot x)_+^2 \Big | \le C \rho ^{1/2} (\tau s)^2 \, . \end{aligned}$$

#### Proof

The first statement is the content of [19, Lemma 6].

To show (5.16), denote $r = \tau s$ and take the obvious candidate $h(x) = \frac{\lambda }{2} (x\cdot e)_+^2$ as a competitor for the infimum. Then $v = u-h$ is harmonic outside of the strip . Let *w* solve  with zero boundary values on $\partial B_r$. It is straightforward to show that $\Vert w \Vert _{L^\infty (B_r)} \le C \rho r^2$. We may now apply, for example, a Three Ball Theorem^1^ to conclude. It states that if *g* is a harmonic function in $B_r$, then, for every $\theta , t \in (0,1)$ such that $B_{t}(y) \subseteq B_{r}$,

$$\begin{aligned} \Vert g \Vert _{L^2(B_{\theta t}(y))} \le \Vert g \Vert _{L^2(B_{\theta ^2 t}(y))}^{1/2} \Vert g \Vert _{L^2(B_{t}(y))}^{1/2}. \end{aligned}$$

We apply this with $g = u-h-w$ and parameters $y = \frac{1}{8}e r$, $\theta = \frac{1}{3}$, $t = \frac{7}{8} r$ to get

$$\begin{aligned} \Vert g \Vert _{\underline{L}^2(B_{s/6})} \le C \Vert g \Vert _{\underline{L}^2(B_{\theta t}(y)) } \le C \Vert g \Vert _{\underline{L}^2(B_{\theta ^2 t}(y))}^{1/2} \Vert g \Vert _{\underline{L}^2(B_{7s/8}(y)) }^{1/2} . \end{aligned}$$

Since *g* is harmonic and $u=h =0$ in $B_{\theta ^2 t}(y)$, we obtain using the supremum bound obtained before for *w* that

$$\begin{aligned} \Vert u-h \Vert _{L^\infty (B_{r/12})} \le \Vert w \Vert _{L^\infty (B_{r/12})} + C \rho ^{1/2} r^2 \le C \rho ^{1/2} r^2 \,. \end{aligned}$$

This completes the proof of (5.16) since $r = \tau s$. $\square$

We are now ready to prove Proposition 5.2.

#### Proof of Proposition 5.2

We will first apply Lemma 5.3. Using (5.9) with (5.5), we obtain that (5.13) is valid for $s = \gamma r/\theta$. Since $\big ( \mathcal {E}(\varepsilon )\big )^\alpha \le \sigma r^2$, then (5.14) is valid by (5.12) for small enough $\sigma$, and we hence find $x_0 \in \varGamma (u)$ in $B_{\gamma r / \theta }$ by Lemma 5.3.

Next, we apply [20, Lemma 6] on the density of the level strip with the parameters $s \in [\gamma r,\gamma ^{-1} r]$ and $h = s^{1/3} \Vert u^\varepsilon - u \Vert _{L^\infty (B_{1/2})}^{1/3}$,

$$\begin{aligned} |( \varLambda (u^\varepsilon ) {\setminus } \varLambda (u) ) \cap B_{s}(x_0) |&\le | \{ 0< u < h^2 \} \cap B_{s}(x_0)| + \frac{1}{h^2} \int _{\varLambda (u^\varepsilon ) \cap B_s(x_0) } u \\&\le C|B_s| \Bigl ( \frac{h}{s} + \frac{1}{h^2} \Vert u^\varepsilon - u \Vert _{L^\infty (B_{1/2})} \Bigr ) \\  &\le C|B_s| \bigl ( \gamma ^{-2} r^{-2} \big ( \mathcal {E}(\varepsilon )\big )^\alpha \bigr )^{1/3} \\&\le C |B_r|( \gamma ^{-2} \sigma )^{1/3} . \end{aligned}$$

Thus, by assuming that $C ( \gamma ^{-2} \sigma )^{1/3} \le 2^{-d-1}$, we deduce the lower bound for the density of $\varLambda (u)$ around the free boundary point $x_0 \in \varGamma (u)$:

$$\begin{aligned} |\varLambda (u) \cap B_s(x_0)|&\ge | \varLambda (u^\varepsilon ) \cap \varLambda (u) \cap B_s(x_0)| \\  &= | \varLambda (u^\varepsilon ) \cap B_{s}(x_0)| - |( \varLambda (u^\varepsilon ){\setminus } \varLambda (u) ) \cap B_{s}(x_0)| \\&\ge 2^{-d-1} \theta |B_s| \,. \end{aligned}$$

Observe then that  solves $\Delta v = \lambda \chi _{v>0}$ in $B_{1/\Lambda }$, $0 \in \varGamma (v)$ and that the above display and (5.5) imply that ${{\,\textrm{md}\,}}(\varLambda (u) \cap B_s) \ge 2^{-d-1} \Lambda ^{-1/2} \theta s$ as well for every $s \in (\gamma r , \gamma ^{-1} r)$. Thus, by choosing $\vartheta = 2^{-d-1} \Lambda ^{-1/2} \theta$ and $\rho = (C^{-1}\delta \theta )^2/ (4\Lambda )$, we find by Lemma 5.4 constants $s_0,\tau \in (0,1)$, both depending only on $(\delta ,\vartheta ,\lambda ,d,\Lambda )$, such that, for every $s \in (0, \gamma s_0 \wedge r]$,

$$\begin{aligned} \inf _{e \in \partial B_1} \sup _{x \in B_{\tau \gamma ^{-1} s/12}} \Big | v(x) - \frac{\lambda }{2} (e\cdot x)_+^2 \Big | \le \frac{\theta ^2}{4 \Lambda } \delta (\tau \gamma ^{-1} s/12 )^2 \, . \end{aligned}$$

Finally, taking $r_0 = \gamma s_0$ so that $r \le r_0$ implies that $\gamma ^{-1} r \le s_0$, changing variables back and taking $\gamma = \tau /(12 \Lambda ^{1/2})$ and $s = r$ above, we deduce that

$$\begin{aligned} \inf _{h \in \mathbb {H}^0} \Vert u^\varepsilon -h \Vert _{L^\infty (B_{r})} \le \frac{\delta }{2} r^2 \,. \end{aligned}$$

Hence (5.10) follows by the triangle inequality by demanding further that the parameter $\sigma$ is so small that $C \sigma ^\alpha \le \frac{\delta }{2}$. The proof is complete. $\square$

### Improvement of Flatness

Next we show that flatness implies large-scale regularity. The flatness assumption does not involve the rescaled solutions, but it helps us to keep track of rescalings, thereby obtaining large-scale regularity of the free boundary.

We employ a flatness improvement argument, following Andersson [2], written for the Signorini problem. The method can be compared to the De Giorgi’s flatness implies regularity theorem for minimal surfaces [31].

For the obstacle problem the argument takes a much easier form, see Lemma 5.5, and it can be found in the lecture notes by Andersson [1] and by Serra [43].

#### Lemma 5.5

Let $\beta ,\gamma \in (0,1)$. There exist constants $r_0 , \delta \in (0,1)$, both depending only on $(\beta ,\gamma ,\lambda ,d,\Lambda )$, such that if *u* is a solution of  in $B_{1}$, the origin is a free boundary point of *u*, that is, $0 \in \varGamma (u)$, and that

5.17 $$\begin{aligned} \inf _{h \in \mathbb {H}} \Vert u - h \Vert _{\underline{L}^2(B_{1/2})} \le \delta , \end{aligned}$$

then there exists $h \in \mathbb {H}^0$ such that, for every $r \in (0,r_0]$,

5.18 $$\begin{aligned} \Vert u -h \Vert _{L^\infty (B_r)} \le \gamma r^{2+\beta } \inf _{h \in \mathbb {H}} \Vert u-h \Vert _{\underline{L}^2(B_{1/2})} . \end{aligned}$$

#### Proof

Without loss of generality, assume that , and hence *u* solves $\Delta u = \lambda \chi _{\{ u>0\}}$. Assume that $0\in \varGamma (u)$. Fix $\beta ,\gamma \in (0,1)$.

Our initial goal is to show that there exist small constants $\delta ,s \in (0,1)$, both depending only on $(\beta ,\gamma ,\lambda , d)$, such that if

$$\begin{aligned} \inf _{(e,t) \in \partial B_1 \times \mathbb {R}} \Vert u - \tfrac{\lambda }{2} (e \cdot x+t)_+^2 \Vert _{L^2(B_{1/2})} \le \delta , \end{aligned}$$

then

5.19 $$\begin{aligned}  &   \inf _{(e,t) \in \partial B_1 \times \mathbb {R}} \Vert u -\tfrac{\lambda }{2} (e \cdot x+t)_+^2 \Vert _{L^\infty (B_{s})} \nonumber \\  &   \quad \le \gamma s^{2+\beta } \inf _{(e,t) \in \partial B_1 \times \mathbb {R}} \Vert u -\tfrac{\lambda }{2} (e \cdot x+t)_+^2 \Vert _{\underline{L}^2(B_{1/2})}. \end{aligned}$$

We argue by contradiction, based on the following elementary argument in Step  1.

*Step 1.* We show that, for every $\gamma \in (0,1)$, there exists a constant $c(\gamma ,d) \in (0,1/2]$ such that if $w \in H^1_{\textrm{loc}}(B_1)$ is the weak solution of the equation

$$\begin{aligned} \Delta w =0 \text { in } B_1^+:=B_1 \cap \{ x_d >0\}, \text { and } w \equiv 0 \text { in } B_1^-:=B_1 \cap \{ x_d <0\} . \end{aligned}$$

and if *w* satisfies the conditions $\int _{B_c} w(x) (x_d)_+ \, \textrm{d}x = 0$ and $\int _{B_c} w(x) x_k (x_d)_+\, \textrm{d}x = 0$ for $k \in \{1,\ldots ,d-1 \}$, then, for every $s \in (0,c]$,

5.20 $$\begin{aligned} \left\| w \right\| _{L^\infty \left( B_{s} \right) } \le \frac{\gamma }{2} s^{2+\beta } \left\| w \right\| _{\underline{L}^2 \left( B_{1} \right) } . \end{aligned}$$

The proof is based on the fact that we find by odd reflection a harmonic function $w'$ in $B_1$, which coincides with *w* in $B_1 \cap \{ x_d >0\}$. By harmonicity, there exists a radius $r(d) \in (0,1)$ such that $w' = \sum _{k=0}^\infty p_k$ in $B_r$, where $p_k$ is a harmonic homogeneous polynomial of degree *k*. Now, the boundary condition $w' = 0$ on $B_1 \cap \{ x_d = 0\}$ dictates that $p_0 = 0$, $p_1(x) = a_0 x_d$ and $p_2(x) = \sum _{k=1}^{d-1} a_k x_k x_d$. Then the orthogonality conditions imply that $p_1 = 0$ and $p_2 = 0$, and that, for every $s \in (0,c]$, $\Vert w \Vert _{L^\infty (B_s)} \le C s^3 \Vert w\Vert _{L^2(B_1)}$. We may then choose *c* small enough so that $c^{1-\beta } 2 C \le \gamma$ and obtain (5.20).

*Step 2.* Fix $\gamma \in (0,1)$ and let $c(\gamma ,d)$ be as in Step 2. We now prove (5.23) via contradiction. We assume, on the contrary, that there exist a sequence $\{u^j\}_j$ solving $\Delta u^j = \lambda \chi _{\{u^j > 0\}}$ with $0 \in \varGamma ({u_j})$ and another sequence $\{h^j\}_j$ with $h^{j}(x) = \tfrac{\lambda }{2} (e^{j} \cdot x+t_{j})_+^2$ such that $\delta _j := \Vert u^j - h^j \Vert _{L^2(B_1)} \rightarrow 0$ as $j\rightarrow \infty$ while

5.21 $$\begin{aligned} \inf _{(e,t) \in \partial B_1 \times \mathbb {R}} \Vert u^j -\tfrac{\lambda }{2} (e \cdot x+t)_+^2 \Vert _{\underline{L}^2(B_{c})} > \gamma c^{2+\beta } \Vert u^j - h^j \Vert _{\underline{L}^2 \left( B_{1} \right) } . \end{aligned}$$

After rotation, assume, up to a relabeled subsequence, that $e^j \rightarrow e_d$ as $j \rightarrow \infty$. Set

5.22 $$\begin{aligned} v^j =\frac{u^j -{h}^j }{\Vert u^j -{h}^j \Vert _{L^2(B_1)} }. \end{aligned}$$

Since both $u^j$ and $h^j$ are nonnegative minimizers of the energy $\int _{B_1}(\tfrac{1}{2} |\nabla w|^2 + \lambda w)$, one actually obtains using admissible competitors that $v^j \Delta v^j \ge 0$ and $\Delta (v^j)^2 \ge 0$ weakly in $B_1$. The latter implies that $\Vert v^j \Vert _{L^\infty (B_{1/2})} \le C(d)$. Using the first one, it is possible to show, up to passing to a subsequence, that there exists $v \in H_{\textrm{loc}}^1(B_1)$ such that $v^j \rightarrow v$ in $H_{\textrm{loc}}^1(B_1)$ as $j \rightarrow \infty$. Now, since $0 \in \varGamma ({u^j})$, we have that $|v^j(0)| = \delta _j^{-1} (t^j)_+^2 \le C$. The non-degeneracy, on the other hand, implies that $(t^j)_-^2 \le C \delta _j$, and thus $t_j \rightarrow 0$ as $j \rightarrow \infty$. Applying Lemma 2.7, we may show that $v^j \rightarrow v = 0$ in $B_1 \cap \{ x_d < 0\}$ (using 2.32) and that $\Delta v = 0$ in $B_1 \cap \{ x_d > 0\}$ (using 2.31). Furthermore, for given $s\in (0,1)$, letting $h^{j,s} = \tfrac{\lambda }{2} (e^{s,j} \cdot x+t^{s,j})_+^2$ be the minimizing half-space solution for $u^j$ in $B_s$ and taking variations with respect to *t* and *e*, we see that, for every $e' \in (e^{s,j})^\perp$,

$$\begin{aligned} \int _{B_s} (h^{j,s})^{1/2} \left( u^j(x)- h^{j,s} \right) \textrm{d}x = 0 = \int _{B_s} (h^{j,s})^{1/2} (e' \cdot x) \left( u^j(x)- h^{j,s} \right) \textrm{d}x . \end{aligned}$$

Since $t_j \rightarrow 0$ and $e^j \rightarrow e_d$ as $j \rightarrow \infty$, the above display yields, after passing to the limit, that $\int _{B_c} v(x) (x_d)_+ \, \textrm{d}x = 0$ and $\int _{B_c} v(x) x_k (x_d)_+\, \textrm{d}x = 0$ for $k \in \{1,\ldots ,d-1 \}$. Thus we are in position to use the result of Step 2 and obtain a contradiction with (5.21) for large enough *j* by the strong convergence in $L^2$.

*Step 3.* Iterating (5.23) and using the sup bound for $(u-h)^2$ gives us that, for every $s \in (0,c]$,

5.23 $$\begin{aligned}  &   \inf _{(e,t) \in \partial B_1 \times \mathbb {R}} \Vert u -\tfrac{\lambda }{2} (e \cdot x+t)_+^2 \Vert _{L^\infty (B_{s})} \nonumber \\  &   \quad \le \gamma s^{2+\beta } \inf _{(e,t) \in \partial B_1 \times \mathbb {R}} \Vert u -\tfrac{\lambda }{2} (e \cdot x+t)_+^2 \Vert _{\underline{L}^2(B_{1/2})}. \end{aligned}$$

Let $e^s$ and $t^s$ realize the minimum on the right and denote $h^s = \tfrac{\lambda }{2} (e^s \cdot x+t^s)_+^2$. Then, by the triangle inequality and simple manipulations,

$$\begin{aligned} s \bigl (s | e^{2s} - e^{s}| + | t^{2s} - t^{s}| \bigr ) \le C \gamma s^{2+\beta } \inf _{(e,t) \in \partial B_1 \times \mathbb {R}} \Vert u -\tfrac{\lambda }{2} (e \cdot x+t)_+^2 \Vert _{\underline{L}^2(B_{1/2})} \end{aligned}$$

Thus both $\{e^s\}_s$ and $\{t^s\}_s$ are Cauchy sequences, and $e^s \rightarrow e$ and $t^s \rightarrow t$ as $s \rightarrow 0$. Since $u(0) = 0$, we must have that $t = 0$, and therefore, from the previous display, $s |e^s - e| + |t^s| \le C \gamma s^{2+\beta } \inf _{(e,t) \in \partial B_1 \times \mathbb {R}} \Vert u -\tfrac{\lambda }{2} (e \cdot x+t)_+^2 \Vert _{\underline{L}^2(B_{1/2})}$. Defining hence $h = \tfrac{\lambda }{2} (e \cdot x)_+^2$ and taking $\gamma$ smaller, we conclude with (5.18). The proof is complete. $\square$

The quantitative estimate above, extends the regularity of the free boundary result to a neighborhood of the origin, i.e let $$ y_0 \in \varGamma (u) \cap B_{1/2}$$, then there exists $$ h^{y_0}$$, such that

$$\begin{aligned} \Vert u_{r,y_0} - h^{y_0} \Vert _{L^2(B_{1/2})} \le C r^\beta \Vert u-h \Vert _{L^2(B_1)}, \end{aligned}$$

and the corresponding unit normal vector $$ e^{y_0}$$ is Hölder continuous;

$$\begin{aligned} | e^{y_0} -e^0 | \le C| y_0 |^\beta , \end{aligned}$$

hence $\varGamma (u) \cap B_{1/2}$ is a $C^{1,\beta }$-surface.

#### Lemma 5.6

Fix $\beta \in (0,1)$. There exist constants $\rho , \omega \in (0,\tfrac{1}{4})$, both depending only on $(\beta ,\lambda ,d,\Lambda )$, such that if $u^\varepsilon$ and *u* solve5.24and if $0 \in \varGamma (u^\varepsilon )$ and

5.25 $$\begin{aligned} \inf _{h \in \mathbb {H}}\Vert u^\varepsilon -h \Vert _{L^\infty (B_{1/2})} + \Vert u^\varepsilon - u \Vert _{L^\infty (B_{1/2})} \le \omega \,, \end{aligned}$$

then

5.26 $$\begin{aligned} \inf _{h \in \mathbb {H}} \Vert u^\varepsilon -h \Vert _{L^\infty (B_\rho )} \le \rho ^{2+\beta } \inf _{h \in \mathbb {H}} \Vert u^\varepsilon -h \Vert _{L^\infty (B_{1})} + 2 \Vert u^\varepsilon - u \Vert _{L^\infty (B_{1/2})} \,. \end{aligned}$$

#### Proof

Fix $\beta \in (0,1)$ and let $\gamma = 2^{-2-\beta }$. Let $r_0,\delta$ be the parameters from Lemma 5.5 corresponding $(\beta ,\gamma )$. Fix $\rho = \frac{1}{2} r_0$. Let $\theta \in (0,1)$ be a small parameter to be fixed and set $s = \theta ^{-1} \omega ^{1/2}$. We assume that $\omega$ is so small that $s \le \rho$. Suppose that $0 \in \varGamma (u^\varepsilon )$ and that $h(x) = \tfrac{\lambda }{2} \frac{ (e \cdot x+t)_+^2}{e \cdot {\overline{\textbf{a}}} e}$ is the member of $\mathbb {H}$ realizing the infimum in (5.25).

We first show that |*t*| in the definition of *h* is small. On the one hand, by non-degeneracy (2.32), we get, for every $r \in (0,1/2)$, that

$$\begin{aligned} c r^2 \le \Vert u^\varepsilon \Vert _{L^\infty (B_{r})} \le \Vert h \Vert _{L^\infty (B_{r})} + \Vert u^\varepsilon - h \Vert _{L^\infty (B_{r})} \le \Vert h \Vert _{L^\infty (B_{r})} + \omega \,. \end{aligned}$$

Therefore, taking $r = 2 (\omega /c)^{1/2}$, we obtain that

$$\begin{aligned} \Vert h \Vert _{L^\infty (B_{r})}> 0 \quad \implies \quad t > - r = - 2 (\omega /c)^{1/2} \ge - C \theta s \,. \end{aligned}$$

On the other hand, since $u^\varepsilon (0) = 0$,

$$\begin{aligned} t_+^2 = | u^\varepsilon (0) -h(0)| \le \Vert u^\varepsilon -h \Vert _{L^\infty (B_{1/2})} \le \omega = \theta ^2 s^2. \end{aligned}$$

Thus, we obtain that $|t| < C\theta s$. Furthermore, by the triangle inequality, we deduce with $h_0 = \tfrac{\lambda }{2} \frac{ (e \cdot x)_+^2}{e \cdot {\overline{\textbf{a}}} e}$ and

$$\begin{aligned} \Vert u - h_0 \Vert _{L^\infty (B_{\rho })} \le \omega + C (\rho +|t|) |t| \le C\theta (\rho + s) s \le C\theta \rho ^2 \, . \end{aligned}$$

For small enough $\theta (\lambda ,d,\Lambda )$ this implies that there exists a point $x_0$ in $\varGamma (u) \cap B_\rho$. Indeed, it is clear that $B_\rho \subseteq \varLambda (u)$ is impossible. On the other hand, if $B_\rho$ would belong to $\{ u>0 \}$, then *u* would solve  in $U_\rho := B_{\rho /2}(- e \rho /2)$ with $\Vert u \Vert _{L^\infty (U_\rho )} \le C \theta \rho ^2$. Then the functionwould be -harmonic in $U_\rho$ and have boundary values less than $C \theta \rho ^2$. Therefore, by the maximum principle, $\Vert v \Vert _{L^\infty (U_\rho )} \le C \theta \rho ^2$. For small enough $\theta$ this would then violate the bound $\Vert u \Vert _{L^\infty (U_\rho )} \le C \theta \rho ^2$. In conclusion, there exists a point $x_0$ in $\varGamma (u) \cap B_\rho$.

We now obtain by Lemma 5.5 that

$$\begin{aligned} \inf _{h \in \mathbb {H}} \Vert u - h \Vert _{L^\infty (B_{\rho })} \le \inf _{h \in \mathbb {H}} \Vert u - h \Vert _{L^\infty (B_{2\rho }(x_0))} \le \rho ^{2+\beta } \inf _{h \in \mathbb {H}} \Vert u - h \Vert _{L^\infty (B_{1/2})}, \end{aligned}$$

and, once again by the triangle inequality,

$$\begin{aligned} \inf _{h \in \mathbb {H}} \Vert u^\varepsilon - h \Vert _{L^\infty (B_{\rho })} \le \rho ^{2+\beta } \inf _{h \in \mathbb {H}} \Vert u^\varepsilon - h \Vert _{L^\infty (B_{1})} + 2 \Vert u^\varepsilon - u \Vert _{L^\infty (B_{1/2})} \,. \end{aligned}$$

The proof is complete. $\square$

#### Proof of Theorem 1.3

Fix $\beta ,\vartheta \in (0,\frac{1}{2})$ and let $\omega ,\rho \in (0,1)$ be as in Lemma 5.6 corresponding $2\beta$. Throughout the proof, for given $t \in (0,1]$, we consider solutions $u_t^\varepsilon = t^{-2} u^\varepsilon (t \cdot )$ and $u_t$ of the equationsSince $0 \in \varGamma (u_t^\varepsilon )$, we have by Lemmas 2.6 and 2.7 that

$$\begin{aligned} \left\| \nabla u_t^\varepsilon \right\| _{L^{2+\alpha } \left( B_1\right) } \le C \Bigl ( \left\| u_t^\varepsilon \right\| _{L^{2} \left( B_2 \right) } + \lambda \Bigr ) \le C. \end{aligned}$$

Thus Theorem 1.1 and Lemmas 2.1 and 2.6 yield that, for every $t \in (0,1)$,

$$\begin{aligned} \Vert u_t^{\varepsilon } - u_t \Vert _{L^\infty (B_{1/2})} \le C \big ( \mathcal {E} \bigl ( \tfrac{\varepsilon }{t} \bigr ) \big )^{\alpha } \,. \end{aligned}$$

Furthermore, since we assume (1.27), we may apply Proposition 5.2 with $\delta = \frac{1}{2} \omega$ and obtain that

$$\begin{aligned} \inf _{h \in \mathbb {H}^0} \Vert u^\varepsilon -h \Vert _{L^\infty (B_{r_0})} \le \frac{\omega }{2} r_0^2 \quad \text{ and } \quad C \big ( \mathcal {E}(\varepsilon )\big )^\alpha \le \frac{1}{2} \bigl ( \omega \wedge \sigma \bigr ) r_0^2 \,. \end{aligned}$$

Therefore, combining the above two displays, yields that

$$\begin{aligned} \inf _{h \in \mathbb {H}} \Vert u_{r_0}^\varepsilon -h_{r_0} \Vert _{L^\infty (B_{1})} + \Vert u_{r_0}^{\varepsilon } - u_{r_0} \Vert _{L^\infty (B_{1})} \le \omega \,. \end{aligned}$$

Lemma 5.6 then gives us

5.27 $$\begin{aligned} \inf _{h \in \mathbb {H}} \Vert u_{\rho r_0}^\varepsilon -h \Vert _{L^\infty (B_\rho )}&\le \rho ^{2\beta } \inf _{h \in \mathbb {H}} \Vert u_{r_0}^\varepsilon -h \Vert _{L^\infty (B_{1})} + 2 \rho ^{-2} \Vert u_{r_0}^\varepsilon - u_{r_0} \Vert _{L^\infty (B_{1/2})} \nonumber \\&\le \rho ^{2\beta } \inf _{h \in \mathbb {H}} \Vert u_r^\varepsilon -h \Vert _{L^\infty (B_{1})} + C \big ( \mathcal {E} \bigl ( \tfrac{\varepsilon }{r_0} \bigr ) \big )^{\alpha } \,. \end{aligned}$$

Setting $r_k = \rho ^k r_0$, $k\in \mathbb {N}$, we can iterate this as long as $m \in \mathbb {N}$ is such that

$$\begin{aligned} 4 C \big ( \mathcal {E} \bigl ( \tfrac{\varepsilon }{r_m} \bigr ) \big )^{\alpha } \le \omega \,. \end{aligned}$$

This condition is guaranteed by the minimal scale condition as long as $r_m \ge R_{\sigma ,\alpha }$, where $\sigma \le (2C)^{-1} \omega$. Indeed, we inductively assume that, for every $k \in \{1,\ldots ,n \}$, we have that

$$\begin{aligned} \inf _{h \in \mathbb {H}} \Vert u_{r_k}^\varepsilon -h \Vert _{L^\infty (B_{1/2})}&\le r_k^{2\beta } \inf _{h \in \mathbb {H}} \Vert u_r^\varepsilon -h \Vert _{L^\infty (B_{1})} + C \sum _{j=0}^{k-1} \big ( \tfrac{r_k}{r_j}\bigr )^{2\beta } \big ( \mathcal {E} \bigl ( \tfrac{\varepsilon }{r_j} \bigr ) \big )^{\alpha } . \end{aligned}$$

Thus we get, by taking $\sigma$ small enough,

$$\begin{aligned} \inf _{h \in \mathbb {H}} \Vert u_{r_k}^\varepsilon -h \Vert _{L^\infty (B_{1/2})}&\le r_k^{2\beta } \inf _{h \in \mathbb {H}} \Vert u_r^\varepsilon -h \Vert _{L^\infty (B_{1})} + C \sum _{j=0}^{k-1} \big ( \tfrac{r_k}{r_j}\bigr )^{2\beta } \big ( \mathcal {E} \bigl ( \tfrac{\varepsilon }{r_j} \bigr ) \big )^{\alpha } \le \frac{\omega }{2} \end{aligned}$$

and, as already observed before,

$$\begin{aligned} \Vert u_{r_k}^{\varepsilon } - u_{r_k} \Vert _{L^\infty (B_{1/2})} \le C \big ( \mathcal {E} \bigl ( \tfrac{\varepsilon }{r_k} \bigr ) \big )^{\alpha } \le \frac{\omega }{2} \,, \end{aligned}$$

and hence we may take an induction step applying Lemma 5.6. In conclusion, we have so far shown, after relabelling constants, that there exist constants $\alpha (d,\Lambda ) \in (0,1)$ and $C(\beta ,\vartheta ,\lambda ,d,\Lambda )<\infty$ such that, for every $r\in [R_{\sigma ,\alpha } ,1]$,

5.28 $$\begin{aligned} \inf _{h \in \mathbb {H}} \Vert u_r^\varepsilon -h \Vert _{L^\infty (B_{1/2})} \le Cr^{2\beta } + C \big ( \mathcal {E} \bigl ( \tfrac{\varepsilon }{r} \bigr ) \big )^{2\alpha } \,. \end{aligned}$$

We then move towards showing the uniform estimate. Letting $h_r(x) = \frac{\lambda }{2} \frac{ ( e_r \cdot x + t_r)_+^2 }{e_r \cdot {\overline{\textbf{a}}} e_r}$ be the realizing the infimum in (5.28), we have that

$$\begin{aligned} |e_{2s} - e_s| + s^{-1} |t_{2s} - t_s|&\le C \inf _{h \in \mathbb {H}} \Vert u_s^\varepsilon -h \Vert _{L^\infty (B_{1/2})} + C \inf _{h \in \mathbb {H}} \Vert u_{2s}^\varepsilon -h \Vert _{L^\infty (B_{1/2})} \\  &\le Cs^{2\beta } + C \big ( \mathcal {E} \bigl ( \tfrac{\varepsilon }{s} \bigr ) \big )^{2\alpha } \,. \end{aligned}$$

This implies that by the condition (1.19) that, for every $s \in [2^k r,2^{k+1}r]$,

$$\begin{aligned}&s^{-1} |t_s - t_r| + |e_s - e_r| \le C r^{2\beta } \sum _{j=0}^k 2^{j2\beta } + C \sum _{j=0}^k \big ( \mathcal {E} \bigl ( \tfrac{\varepsilon }{2^j r} \bigr ) \big )^{2\alpha } \\&\quad \le C s^{2\beta } + C \big ( \mathcal {E} \bigl ( \tfrac{\varepsilon }{r} \bigr ) \big )^{2\alpha } \,. \end{aligned}$$

Furthermore, similarly to the proof of Lemma 5.6, we also obtain that

$$\begin{aligned} |t_r| \le Cr^{1+\beta } + C r \big ( \mathcal {E} \bigl ( \tfrac{\varepsilon }{r} \bigr ) \big )^{\alpha } \,. \end{aligned}$$

Using these we obtain that, for $h^{(r)} = \frac{\lambda }{2} \frac{ ( e_r \cdot x)_+^2 }{e_r \cdot {\overline{\textbf{a}}} e_r}$

$$\begin{aligned} \Vert h_{r} - h_s\Vert _{L^\infty (B_s)} \le C s^2 \Bigl (s^{2\beta } + r^{\beta } + \big ( \mathcal {E} \bigl ( \tfrac{\varepsilon }{s} \bigr ) \big )^{2\alpha } + \big ( \mathcal {E} \bigl ( \tfrac{\varepsilon }{r} \bigr ) \big )^{\alpha } \Bigr ) \,. \end{aligned}$$

Combining this with (5.28) then concludes the proof. $\square$

We finally remark that if the minimal scale is independent of the reference point, that is, for every $x \in \mathbb {R}^d$ we have

5.29 $$\begin{aligned} \mathcal {E}( \varepsilon , x) \le \mathcal {E}( \varepsilon ) \qquad \forall x \in \mathbb {R}^d, \end{aligned}$$

then we will obtain large-scale regularity of the free boundary. Notice that (5.29) is valid, for example, in the case of periodic or almost periodic homogenization.

#### Theorem 5.7

Assume that (5.29) is valid. Let $u^\varepsilon$ be a solution to the heterogenous obstacle problem, with $f \equiv \lambda >0$. Assume that $0 \in \varGamma (u^\varepsilon )$, and $\inf _{h \in \mathbb {H}} \Vert u^\varepsilon - h \Vert _{L^2(B_2)} =\delta$ is small enough, then $\varGamma (u^\varepsilon ) \cap B_{1/2}$ is large-scale $C^{1,\beta }$-regular, in the sense that every $x_0 \in \varGamma (u^\varepsilon ) \cap B_{1/2}$ is a large-scale regular free boundary point, (see Definition 1.4), and (1.28) holds at $x_0$.

#### Proof

The Lebesgue measure of the set $$ \varLambda (u^\varepsilon _r) \cap B_1 $$ can be estimated from below, employing Lemma 5.6. Without loss of generality, let us assume that the half-space solution *h* in the Lemma 5.6 depends on direction $$ e=e_d$$. Hence

5.30 $$\begin{aligned} \Vert u^\varepsilon _{r} \Vert _{L^\infty (B_1 \cap \{ x_d<0\} )} \le C r^\beta \delta +C \mathcal {E}(\tfrac{\varepsilon }{r} )^{\alpha } . \end{aligned}$$

Now let $x^0 \in B_1 \cap \{ x_d<0\}$ be such that $u_r^\varepsilon (x^0)>0$, and let $2 \tau :=- x_d^0=-x^0 \cdot e_d>0$, then by the non-degeneracy of $u_r^\varepsilon$,

$$\begin{aligned} c \tau ^2 \le \sup _{B_\tau (x_0)} u^\varepsilon _r \le C r^{\beta } \delta +C \mathcal {E}(\tfrac{\varepsilon }{r} )^{\alpha }. \end{aligned}$$

Therefore we obtain

$$\begin{aligned} \tau ^{2} \le C r^\beta \delta +C (\mathcal {E} (\tfrac{\varepsilon }{r} ))^{ \alpha }. \end{aligned}$$

Hence,

$$\begin{aligned} | \{ u_r^\varepsilon >0\} \cap \{ x_d <0 \} | \le c_d \tau \end{aligned}$$

is small, and

$$\begin{aligned} \frac{| \varLambda ( u^\varepsilon _r) \cap B_1| }{|B_1|} \ge \frac{1}{2}-C\tau>\vartheta >0, \end{aligned}$$

if $r\ge R_{\sigma ,\alpha }$. Applying Theorem 1.3, we obtain (1.28).

Now if $x_0 \in \varGamma (u^\varepsilon ) \cap B_{1/2}$, then we can apply Lemma 5.6 and Theorem 1.3 for the rescaled solutions $u^\varepsilon _{r, x_0}$. $\square$

## Data Availability

Data sharing not applicable to this article as no datasets were generated or analysed during the current study.

## Examples in Dimension One

In this appendix we briefly discuss homogenization of the obstacle problem and its free boundary in dimension one. We give two important one-dimensional examples, which clarify the main assumptions and the setting of our problem.

### Homogenization of the Normalized Obstacle Problem and Its Free Boundary in 1D

Let the coefficient field $\textbf{a}$ be a continuous function taking values in [1, 2]. Let $u_\varepsilon \ge 0$ be the solution of the following one-dimensional obstacle problem, with oscillating coefficients;

$$\begin{aligned} \left\{ \begin{aligned}&(a(\tfrac{\cdot }{\varepsilon })u^\prime _\varepsilon )^\prime =\chi _{\{u_\varepsilon >0\}} \quad \text{ in } (0,4), \\&u_\varepsilon (0)=0, \; u_\varepsilon (4)= 1. \end{aligned} \right. \end{aligned}$$

Denote by

$$\begin{aligned} \alpha _\varepsilon :=\inf \{x \in (0,4) \, : \, u_\varepsilon (x)>0\} \end{aligned}$$

the free boundary point of the problem. This is a unique point since, by the minimum principle, $u_\varepsilon$ is positive in $(\alpha _\varepsilon , 4)$ and zero on $[0,\alpha _\varepsilon ]$. After a direct computation, the solution can be written as

A.1 $$\begin{aligned} u_\varepsilon (x) = \int _{\alpha _\varepsilon }^{x \wedge \alpha _\varepsilon } (t - \lambda _\varepsilon ) a^{-1}(\tfrac{t}{\varepsilon })\, \textrm{d}t , \quad \end{aligned}$$

where $\lambda _\varepsilon$ is chosen as

A.2 $$\begin{aligned} \lambda _\varepsilon = \Big ( \int _{\alpha _\varepsilon }^{4} t a^{-1}(\tfrac{t }{\varepsilon })\, \textrm{d}t - 1 \Big ) \Big ( \int _{\alpha _\varepsilon }^{4} a^{-1}(\tfrac{t}{\varepsilon })\, \textrm{d}t \Big )^{-1} \end{aligned}$$

to guarantee the boundary condition $u_\varepsilon (4)=1$. Since *a* takes values less than 2, we see that $\lambda _\varepsilon$ is positive. Employing then the fact that $u_\varepsilon$ is $C^1$ in the interior (0, 2), we also obtain that $u_\varepsilon ^\prime (\alpha _\varepsilon )=0$, which gives us $\alpha _\varepsilon = \lambda _\varepsilon$ and thus (A.2) gives us the equation to solve $\alpha _\varepsilon$.

Next, we let ${\overline{a}}_\varepsilon$ to be the coarsened coefficient and $\phi _\varepsilon$ the corrector:

$$\begin{aligned} {\overline{a}}_\varepsilon = \Big ( \frac{1}{4} \int _0^4 a^{-1}(\tfrac{x}{\varepsilon }) \, \textrm{d}x \Big )^{-1} \quad \text{ and } \quad \phi _\varepsilon (x) = {\overline{a}}_\varepsilon \int _0^x a^{-1}(\tfrac{t}{\varepsilon }) \, \textrm{d}t - x . \end{aligned}$$

We assume the bound

A.3 $$\begin{aligned} | {\overline{a}}_\varepsilon - {\overline{a}} | + \Vert \phi _\varepsilon \Vert _{L^2(0,4)} \le \mathcal {E}(\varepsilon ) \end{aligned}$$

and $$\mathcal {E}(\varepsilon ) \rightarrow 0$$ as $$\varepsilon \rightarrow 0$$.

Now let

A.4 $$\begin{aligned} \overline{a}:=\mathbb {E} \left( \int _0^1 a^{-1}(t)\textrm{d}t \right) ^{-1}, \end{aligned}$$

be the homogenized matrix, and $\overline{u}$ be the solution of the obstacle problem solves

$$\begin{aligned} \left\{ \begin{aligned}&({\overline{a}} \, {\overline{u}}^\prime )^\prime =\chi _{\{{\overline{u}}>0\}} \quad \text{ in } (0,4), \\&{\overline{u}}(0)=0, \; {\overline{u}}(4)= 1. \end{aligned} \right. \end{aligned}$$

As above, a straightforward computation yields that

$$\begin{aligned} {\overline{u}} (x) = {\overline{a}}^{-1} \int _{{\overline{\alpha }}}^{x \wedge {\overline{\alpha }}} (t - {\overline{\alpha }}) \, \textrm{d}t , \quad \text{ with } \quad {\overline{\alpha }} = 4 - \sqrt{2 {\overline{a}} } \end{aligned}$$

We will then verify that

1.  $$\alpha _\varepsilon \rightarrow \overline{\alpha }$$ a.s. as $$\varepsilon \rightarrow 0$$,
2.  $$u_\varepsilon \rightarrow \overline{u}$$ a.s. as $$\varepsilon \rightarrow 0$$.

Recall that $\alpha _\varepsilon$ and $\overline{\alpha }$ are determined by the following equations:

$$\begin{aligned} \int _0^4 (x-\alpha _\varepsilon )_+a^{-1}(\tfrac{x}{\varepsilon })\textrm{d}x=1 \quad \text {and} \quad \int _0^4 (x-\overline{\alpha })_+\overline{a}^{-1}\textrm{d}x=1. \end{aligned}$$

On the one hand, we have

$$\begin{aligned} \int _0^4 (x-\overline{\alpha })_+\overline{a}^{-1}\textrm{d}x = \frac{1}{2} {\overline{a}}^{-1} (4-{\overline{\alpha }})^2 . \end{aligned}$$

By integration by parts, we obtain

$$\begin{aligned} \int _0^4 (x-{\alpha _\varepsilon })_+ a^{-1}(\tfrac{x}{\varepsilon }) \, \textrm{d}x = \frac{1}{2} {\overline{a}}_\varepsilon ^{-1} (4-{\alpha _\varepsilon })^2 - {\overline{a}}_\varepsilon ^{-1} \int _{\alpha _\varepsilon }^4 \phi (x) \, \textrm{d}x . \end{aligned}$$

Therefore, it follows, after some manipulations, that

$$\begin{aligned} {\overline{\alpha }} - \alpha _\varepsilon = \frac{1}{8 - {\overline{\alpha }} - \alpha _\varepsilon } \biggl ( {\overline{a}}_\varepsilon ({\overline{a}}^{-1} - {\overline{a}}_\varepsilon ^{-1}) + 2 \int _{\alpha _\varepsilon }^4 \phi _\varepsilon (x) \, \textrm{d}x \biggr ) \end{aligned}$$

The assumption (A.3) then implies that

$$\begin{aligned} | {\overline{\alpha }} - \alpha _\varepsilon | \le C \mathcal {E}(\varepsilon ) . \end{aligned}$$

Therefore $\alpha _\varepsilon \rightarrow \overline{\alpha }$. Using the explicit formulas for $u_\varepsilon$ and for $\overline{u}$, together with the convergence $\alpha _\varepsilon \rightarrow \overline{\alpha }$, we obtain the convergence $u_\varepsilon \rightarrow \overline{u}$. Furthermore, the calculations show that in the periodic case,

$$\begin{aligned} \Vert u_\varepsilon -\overline{u} \Vert _{L^\infty } \le C \varepsilon \text { and } | \alpha _\varepsilon -\overline{\alpha } | \le C \varepsilon . \end{aligned}$$

### The Homogenization of the Free Boundary Does not Hold with a Fixed Obstacle

We next show an example demonstrating the instability of the free boundary in homogenization with a fixed obstacle. Indeed, one cannot expect homogenization to hold even if the coefficients are smooth.

To this end, let $\varphi ~$Be a given concave function in the interval $(-1,1)$, called an obstacle, we study the solution to the one-dimensional obstacle problem

A.5 $$\begin{aligned} u \ge \varphi ,~ (\overline{a} u^\prime )^\prime \le 0, \text { and } ~ (\overline{a} u^\prime )^\prime = 0 \text { in } \{ u > \varphi \}. \end{aligned}$$

and the solutions $u_\varepsilon$ to corresponding heterogenous obstacle problem

A.6 $$\begin{aligned} u_\varepsilon \ge \varphi ,~ ({a^\varepsilon } u_\varepsilon ^\prime )^\prime \le 0, \text { and } ~ ({a_\varepsilon } u_\varepsilon ^\prime )^\prime = 0 \text { in } \{ u_\varepsilon > \varphi \}, \end{aligned}$$

with boundary condition $u^\varepsilon (x)=u(x)$ when $x=\pm 1$.

In order to simplify our calculations, we can take $\varphi =\frac{1}{2} -{x^2}$, and the boundary conditions $u^\varepsilon (\pm 1)=u(\pm 1)=0$.

By (A.5), *u* is a linear function in every connected component of the set $\{ u > \varphi \}$, furthermore, if $\overline{x}$ is the free boundary point closest to the boundary point $-1$, then by straightforward calculations, $u(x)= \varphi ^\prime (\overline{x}) (x-\overline{x}) + \varphi {(\overline{x})}$ in the interval $(-1, \overline{x})$ (The first order Taylor polynomial of $\varphi$ at $\overline{x}$). Letting $u(-1)=0$, we obtain $\overline{x}=-1+\frac{\sqrt{2}}{2}$.

Now let us see what similar calculations give for $u_\varepsilon$. Denote by $x_\varepsilon$ the free boundary point closest to the boundary point $-1$, then

$$\begin{aligned} (a(\tfrac{\cdot }{\varepsilon })u^\prime _\varepsilon )^\prime =0 \text { in the interval } (-1,x_\varepsilon ), \end{aligned}$$

and at the free boundary point $x_\varepsilon$ we get

$$\begin{aligned} u_\varepsilon ( x_\varepsilon )=\varphi (x_\varepsilon ), ~ u^\prime _\varepsilon ( x_\varepsilon )=\varphi ^\prime (x_\varepsilon ), \end{aligned}$$

Hence

$$\begin{aligned} u_\varepsilon (x )=a(\tfrac{x_\varepsilon }{\varepsilon }) \varphi ^\prime (x_\varepsilon ) \int _{x_\varepsilon }^x a^{-1}(\tfrac{t}{\varepsilon })\textrm{d}t +\varphi ( x_\varepsilon ), \text { in the interval } (-1,x_\varepsilon ). \end{aligned}$$

Letting $u_\varepsilon (-1)=0$, we get

A.7 $$\begin{aligned} x_\varepsilon a(\tfrac{x_\varepsilon }{\varepsilon }) \int ^{x_\varepsilon }_{-1} a^{-1}(\tfrac{t}{\varepsilon })dt +\frac{1}{2}- x_\varepsilon ^2=0. \end{aligned}$$

Assuming that $x_\varepsilon \rightarrow \overline{x}$ as $\varepsilon \rightarrow 0$, we get a contradiction in (A.7), due to the oscillation of $a(\tfrac{x_\varepsilon }{\varepsilon })$. Hence no homogenization of free boundary result can be expected given a fixed obstacle $\varphi$. This happens, since we have no control over $\nabla \cdot (\textbf{a}^\varepsilon \nabla \varphi )$ as $\varepsilon \rightarrow 0+$. This example fully justifies the normalization of the right hand side in the context of homogenization of the obstacle problem.

## Footnotes

1 The Three Ball Theorem follows from Almgren’s monotonicity formula, that is, $r \mapsto r \Vert \nabla u \Vert _{L^2(B_r)}^2 \Vert u \Vert _{L^2(\partial B_r)}^{-2}$ is non-decreasing.
